# How and why do financial incentives contribute to helping people stop smoking? A realist review

**DOI:** 10.1186/s12889-024-17967-3

**Published:** 2024-02-16

**Authors:** Rikke Siersbaek, Paul Kavanagh, John Ford, Sara Burke, Sarah Parker

**Affiliations:** 1 Centre for Health Policy and Management, Trinity College Dublin, 3-4 Foster Place, Dublin, Ireland; 2 Health Intelligence, Strategic Planning and Transformation, 4th Floor, Jervis House, Jervis Street, Dublin, Ireland; 3 Wolfson Institute for Population Health, Queen Mary University, Charterhouse Square, EC1M 6BQ London, UK

**Keywords:** Smoking cessation, Motivation, Behavior therapy/economics, Behavior therapy/methods, Smoking/therapy

## Abstract

**Background:**

Tobacco smoking remains a key cause of preventable illness and death globally. In response, many countries provide extensive services to help people to stop smoking by offering a variety of effective behavioural and pharmacological therapies. However, many people who wish to stop smoking do not have access to or use stop smoking supports, and new modes of support, including the use of financial incentives, are needed to address this issue. A realist review of published international literature was undertaken to understand how, why, for whom, and in which circumstances financial incentives contribute to success in stopping smoking for general population groups and among pregnant women.

**Methods:**

Systematic searches were undertaken from inception to February 2022 of five academic databases: MEDLINE (ovid), Embase.com, CIHAHL, Scopus and PsycINFO. Study selection was inclusive of all study designs. Twenty-two studies were included. Using Pawson and Tilley’s iterative realist review approach, data collected were screened, selected, coded, analysed, and synthesised into a set of explanatory theoretical findings.

**Results:**

Data were synthesised into six Context-Mechanism-Outcome Configurations and one overarching programme theory after iterative rounds of analysis, team discussion, and expert panel feedback. Our programme theory shows that financial incentives are particularly useful to help people stop smoking if they have a financial need, are pregnant or recently post-partum, have a high threshold for behaviour change, and/or respond well to external rewards. The incentives work through a number of mechanisms including the role their direct monetary value can play in a person’s life and through a process of reinforcement where they can help build confidence and self-esteem.

**Conclusion:**

This is the first realist review to synthesise how, why, and for whom financial incentives work among those attempting to stop smoking, adding to the existing evidence demonstrating their efficacy. The findings will support the implementation of current knowledge into effective programmes which can enhance the impact of stop smoking care.

**PROSPERO registration number:**

CRD42022298941.

**Supplementary Information:**

The online version contains supplementary material available at 10.1186/s12889-024-17967-3.

## Background

Tobacco smoking continues to cause preventable illness and death globally on a huge scale [1] but stopping smoking reduces the occurrence of poor health outcomes [2]. The effectiveness of behavioural and pharmacological components of stop smoking care is well-established, [3, 4] and offering help to stop smoking is an evidence-based tobacco control policy integral to the World Health Organization’s “MPOWER” model [5]. Despite this, even though intention to stop is high among people who smoke, many do not have access to or use stop smoking supports [6–10].

Effective implementation of tobacco control measures has enabled many more developed countries to move into the later stages of the tobacco epidemic, but progress is leaving some population groups behind as smoking prevalence among deprived populations remain higher than among those with more means [11, 12]. For example, in Ireland, while the prevalence of smoking in adults over all reduced from 23% in 2015 to 18% in 2021, the difference in smoking prevalence across socio-economic groups widened across the period from two-fold to almost four-fold [13–15]. In 2015, 16.2% of people in the highest socioeconomic group smoked, a percentage that dropped to 10.6% in 2021. Meanwhile, in 2015, 28.7% of people in the lowest socio economic group smoked, a percentage that rose to 30.7% in 2021 [14]. There is a need not only to strengthen support to help people stop smoking, but also to find approaches that target and tailor support to the needs of population groups with higher smoking prevalence in order to address smoking-related health inequalities [16].

A growing body of evidence shows that financial incentives are effective at helping people stop smoking. A recent Cochrane review by Notley et al. [17] has demonstrated their effectiveness at both helping general populations to stop smoking and to remain abstinent. A separate Cochrane review by Chamberlain et al. of psychosocial interventions to support pregnant women seeking to stop smoking showed that financial incentives are efficacious for that particular population as well [18]. However, while their effectiveness has been established, less is known about how, why, for whom, and to what extent financial incentives work [17]. The challenge now is to find a path from evidence to implementation and ultimately better outcomes especially for those of the greatest need. As Miranda et al. argue, there is a dearth of details published about how to implement financial incentives and ‘the reporting of this information is essential to foster its use’ [19].

Financial incentives have been used in a number of different ways including in the form of direct payments, vouchers, and deposits of a person’s own money which they get back if they stop smoking. And incentives have been given in different amounts, at different intervals during a stop smoking attempt, and with different stipulations and monitoring attached [20].

As a complex addition to an already complex intervention, claims about the direct efficacy of financial incentives have to be considered carefully. Prior studies have shown that financial incentives fundamentally alter a stop smoking intervention by increasing the frequency and quality of the interactions, including ongoing biochemical verification, between a person who is endeavouring to stop smoking and the service. Ormston et al. argue:

Attempting to separate out the effect of the incentive would have been inappropriate and misleading as other inherently linked elements, notably the CO tests, were also key. Mantzari et al. [31] have argued (2012) ‘we need to be cautious about attributing the effects of financial incentives schemes to incentives per se’ as they might operate through various pathways [21].

It is therefore reasonable to assume that financial incentives impact on a stop smoking attempt in several ways: they may play an indirect role in promoting increased engagement with services as well as a direct role through their own immediate monetary impact [21, 22].

For policy makers and practitioners seeking to enhance stop smoking support with financial incentives, while current evidence on efficacy is useful, there is a need to better understand how and in what ways they work so that effective implementation can be planned and evaluated. Using a realist approach we can theorise about the demi-regular patterns of behaviour which are sparked in particular contexts when a financial incentive is introduced in a stop smoking programme to provide the kind of information Miranda et al. call for as discussed above [19].

To progress existing knowledge and provide new evidence for policy makers, we undertook a realist review to answer the questions of how, why, in what circumstances, and for whom financial incentives improve the success of stop smoking interventions among general population groups and among pregnant women [23]. Rather than determining whether an intervention works, the realist approach in the school of Pawson and Tilley seeks to uncover patterns of causality in complex social interventions where they cannot readily be measured or observed [24–26]. Along with synthesising the evidence the study also provides recommendations for how to best use financial incentives in efforts to promote smoking cessation. This study is part of a wider research programme to inform implementation of financial incentives in stop smoking services in Ireland [27].

## Methods

**Fig. 1:**
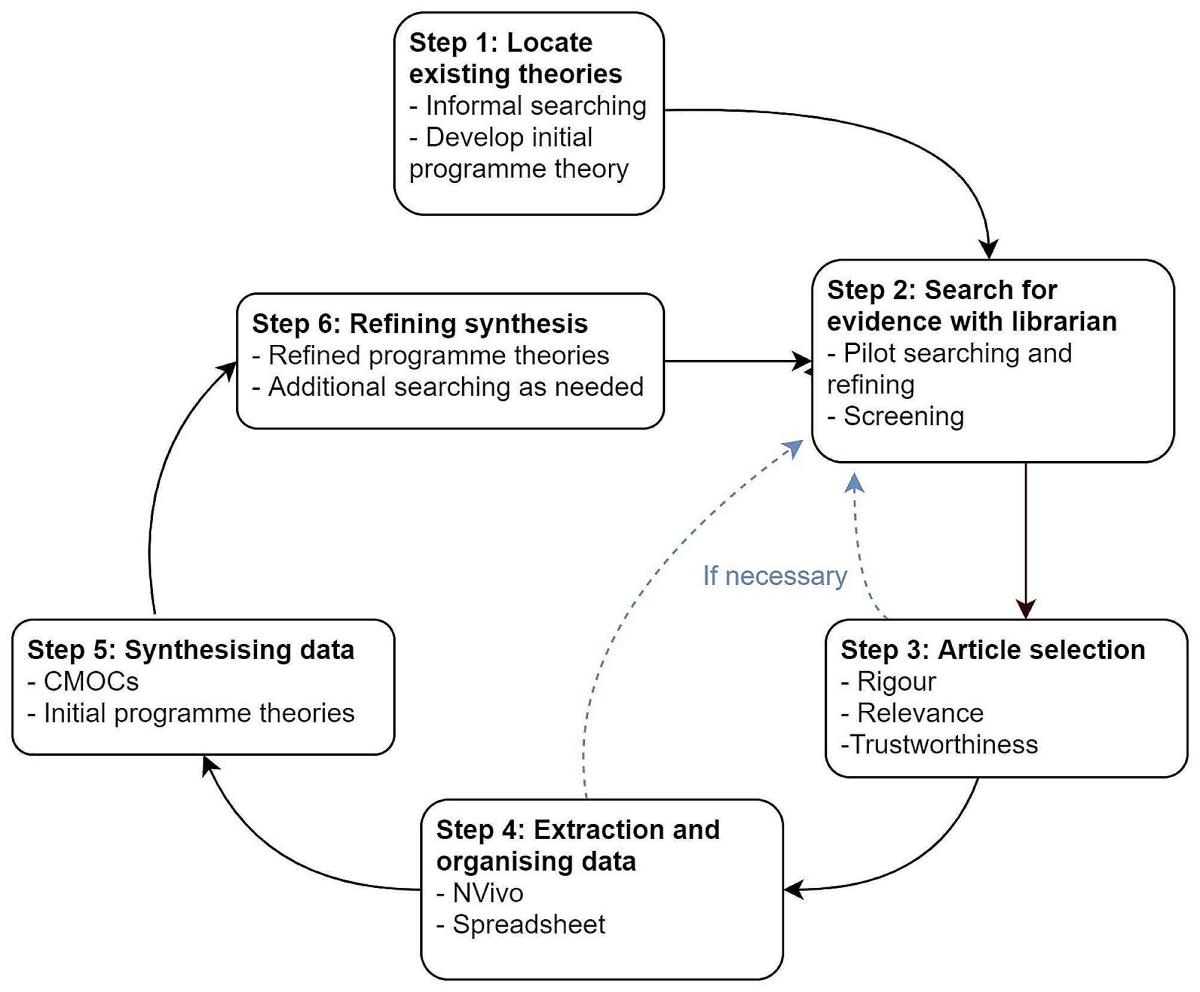
Stages of a realist review

We undertook a realist review following the six-stage, iterative approach (see Fig. 1) detailed by Pawson and Tilley [24–26]. We also were guided by the RAMESES publishing standards in the review and writing process [28].

Realist research in the school of Pawson and Tilley is an explanatory, theory driven approach to understanding complex, social, and open-ended interventions and areas of study. The approach uncovers causation in areas of study where it cannot be readily measured but where data are used to build theories about underlying, hidden powers which operate in the world to produce observable outcomes. Theories are built using multiple pieces of data from several sources, secondary data in the case of realist review and primary data in realist evaluation, and moving from theorising at a level close to the data explaining occurrences in the particular studies under consideration and then moving to higher, more generalisable levels of abstraction through iterative rounds of theory building. Typically realist research provides explanations of causality using the heuristic of context-mechanism-outcome configurations (or CMOCs) to which demonstrate how a particular causal force (a mechanism) is triggered in a given context to produce an outcome [24, 26, 29–32].

**Fig. 2:**
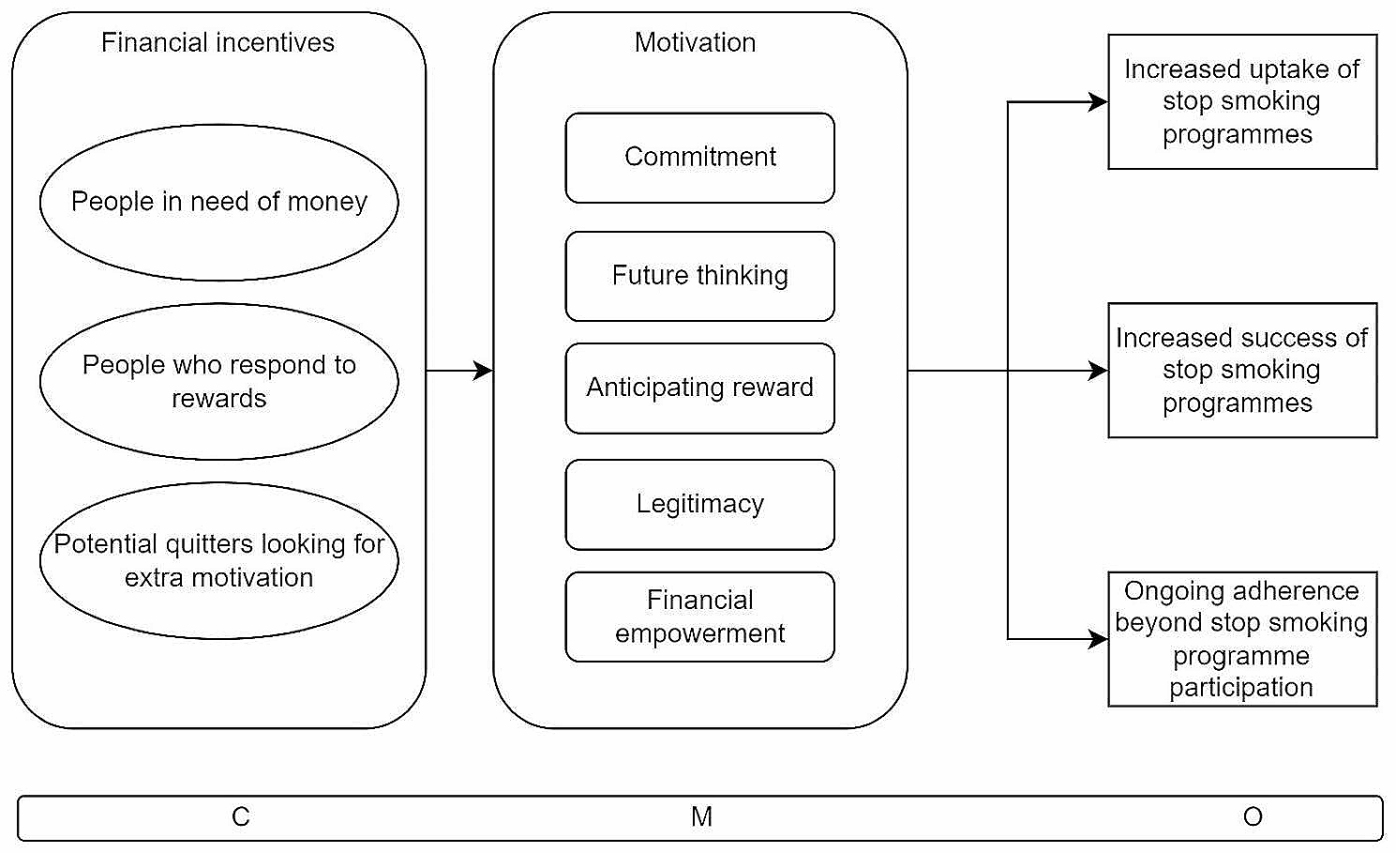
Initial programme theory

In December 2021 we began informal reading and development of an initial programme theory (Fig. 2) which we then used to guide our search strategy. As a result of our initial planning for the study, we registered the study on Prospero (CRD42022298941) and published a study protocol which contains more details about the initial programme theory [23]. Between the time of the Prospero registration and the publication of the study protocol, some changes were made to the search strategy (switching one database for another and refinement of search terms) after consultation with a subject librarian. The full details of the systematic search are described in the additional file.

### Search strategy and databases

Search terms and appropriate databases were identified by the research team in consultation with a subject librarian in Trinity College Dublin. Searches covered dates from inception to February 2022. The terms were selected to generate hits focused on the particular interaction of financial incentives and stopping smoking efforts rather than looking at various stop smoking programmes more broadly because our focus is on how and why financial incentives work or not when added to or used in place of usual stop smoking interventions.

Articles were also identified through recommendations of subject matter experts both on the research team and our expert panel, made up of Irish policy makers, researchers, and health service managers all with significant experience in the stop smoking field, which met with us twice and supplied informal inputs at other times during the review process.

Further iterative searching was not necessary as the above methods of data collection yielded sufficient information to build, test, and refine theory.

### Eligibility criteria

In line with guidance for realist reviews [28] and the aims of this research, inclusion criteria were:

- High quality and relevant articles of any type or design specifically focused on the impact of financial incentives on stop smoking efforts.
- Any year of publication.
- Any general population of interest.
- Any country.
- Written in English language.

Exclusion criteria were:

- Studies that did not explore the direct relationship between financial incentives and stopping smoking.
- Studies which would not add useful data to a realist study. For example, study protocols where the described research was only prospective were excluded because they did not provide data about how the intervention worked, for whom, and why. Similarly, conference abstracts generally were excluded due to their brevity and lack of causal explanations.

There were a variety of excluded studies, for example:

- Studies focused on clinical outcomes in a dependent child or a new-born baby after their mother had given up smoking. These were excluded as the focus of our study was on how the financial incentive influenced the person attempting to give up smoking not the health outcomes of the people around them.
- Cost effectiveness studies of stop smoking programmes using financial incentives.
- Studies examining financial incentives offered with the aim to change behaviour in clinicians providing stop smoking services and not in the person undertaking the stop smoking attempt.
- Studies focusing on neurological aspects of smoking.
- Studies examining other modes of nicotine delivery than tobacco smoking such as vaping.
- Studies focused on a very particular population e.g. people with severe mental illness, people who use drugs, and people with head and neck cancer where the mechanisms driving behaviour might be particular to the experience of that population and not generalisable to broader population groups.

### Data extraction and coding

**Fig. 3:**
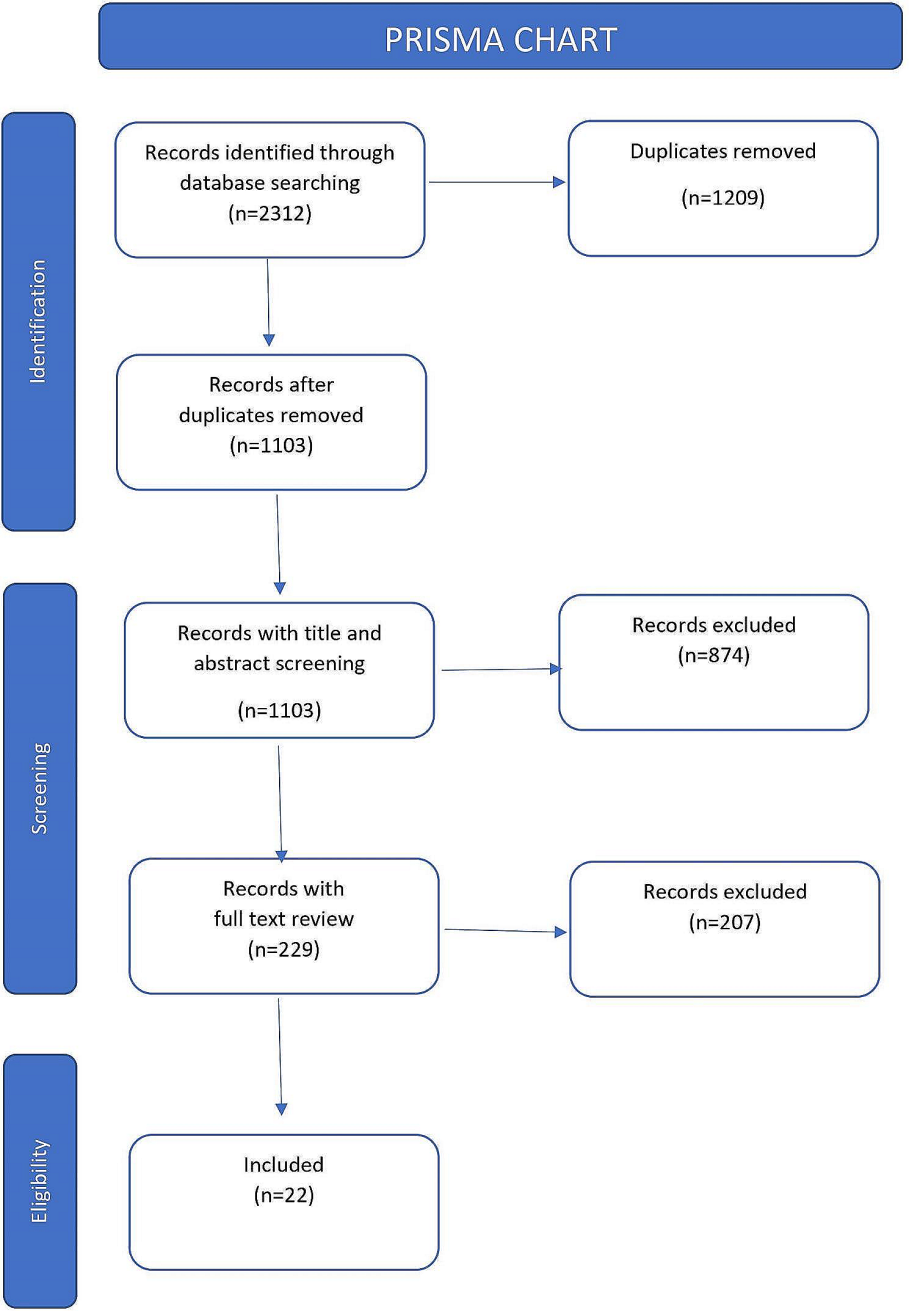
PRISMA chart

The results of the five searches were exported into EndNote 20 and automatic deduplication took place. One researcher (RS) screened the titles and abstracts of all and those which met inclusion criteria were brought forward for full text screening. A second researcher (SP) reviewed 10% of the records for consistency. Conflicts were resolved through discussion. During full text screening articles were also evaluated for rigour and relevance. See the full search in the PRISMA diagram (Fig. 3).

Included articles were imported into NVivo 1.6.1 where inductive, deductive and retroductive coding took place in accordance with the realist approach in the manner described by Papoutsi et al. [33] and Tierney et al. [34]. Accordingly, the data were initially coded inductively into broad descriptive categories with subsequent data coded deductively using codes already created over the course of the coding process. Later, retroductive coding took place to begin to assign context, mechanism, and outcome labels to pieces of data This happened in concert with the initial analytical phase of the study as the relationships between various pieces of data were explored and recorded [7].

Initial broad causal patterns were detected and these were presented to the full research team for robust discussion. This discussion resulted in a focused approach for the next step to explain the direct causal interaction between financial incentives and stopping smoking. As a result, parts of the coded data were excluded from the synthesis because they were not directly engaging with the specific interaction between stop smoking efforts and financial incentives but rather with indirect effects of financial incentives or features of broader stop smoking programmes. While these are important findings they were not the focus of this review.

### Synthesis

The next step was for the lead researcher to craft Context-Mechanism-Outcome configurations (CMOCs) from the subset of the data that directly was related to both financial incentives and smoking cessation. These were then reviewed and discussed again by the full research team and the expert panel, who provided valuable input that challenged and supplemented aspects of the analysis which supported the early development and later refinement of the findings. As a result the data were iteratively combined into six consolidated CMOCs and finally into one overarching programme theory.

## Results

### Included studies

A total of 1103 individual records resulted from the initial five database searches. Titles and abstracts were screened and next 229 underwent full text screening. Subsequently 22 records were selected for inclusion in the review. A number of key papers were suggested by members of the research team and the expert panel such as the Cochrane review by Notley et al. [17] and research arising from the Scottish Give it up for Baby [35] and quit4u [21] studies. These were all picked up by the database search which provided reassurance about the appropriateness of the search terms employed. See the full search in the PRISMA diagram (Fig. 3). A table containing and overview of titles, authors and key features of each article can be found in the supplemental materials file.

### CMOCs

From the data identified as useful and coded in the 22 included articles, 15 initial CMOCs were constructed. These were next consolidated into six CMOCs which were concerned with similar contexts and/or mechanisms, and finally the six consolidated CMOCs were put together into one overarching programme theory. Each of the six consolidated CMOCs are explained below before we then describe the overarching programme theory. See data used to construct CMOCs in supplemental file.

### CMOC1

When a person who smokes also experiences financial insecurity (C), a financial incentive encourages them to initiate a stop smoking attempt and to engage with the wider stop smoking programme because it offers an opportunity to meet financial needs, gives a sense of autonomy due to having extra financial resources, and engenders feelings of esteem and respect (M). This leads to more success in enrolling and maintaining people in stop smoking programmes (O).

**Fig. 4:**
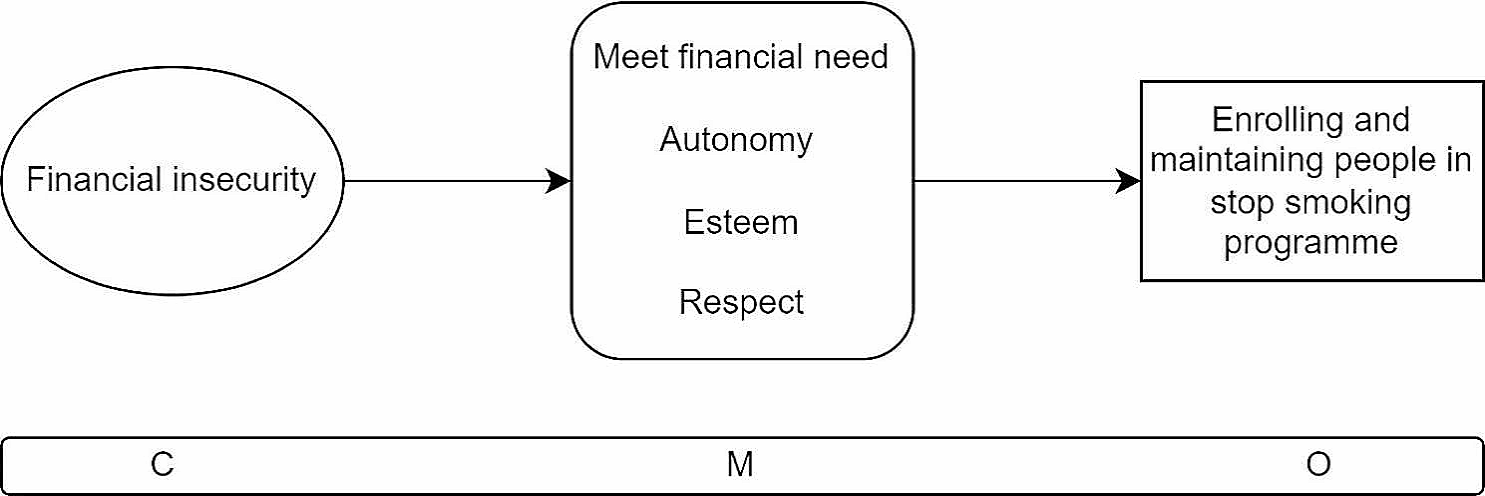
CMOC1

The first CMOC (Fig. 4) explains what happens in a context where a person who smokes also is experiencing financial insecurity. In this context, the financial incentive motivates people to take up a stop smoking attempt because the payment offered triggers the mechanism of experiencing a sense of autonomy by giving them the opportunity to meet financial needs in their lives such as pay bills or to have something extra to spend on discretionary or enjoyable items or activities [20, 35–40].

The context also triggers further mechanisms when people have begun a stop smoking attempt. Here the financial incentive keeps them engaged by an experience of autonomy through the added financial resources accessed and through experiencing feelings of being respected and shown esteem because they are better able to meet their needs through the financial incentive [38, 39, 41, 42]. They also feel valued and trusted to take positive actions by stop smoking counsellors who they have more frequent engagement with than in other stop smoking programme modalities due to ongoing carbon monoxide testing [22, 39, 41, 43]. Altogether these mechanisms lead to the outcome of ongoing engagement with a stop smoking programme [17, 21, 44].

### CMOC2

When people who currently smoke have a high threshold for behaviour change and need tangible and external rewards to initiate change (C), a financial incentive encourages action through the presence of repeated external reinforcement via a payment and offers an opportunity for utilitarian and/or enjoyable spending (M) to initiate and maintain a stop smoking attempt (O).

**Fig. 5:**
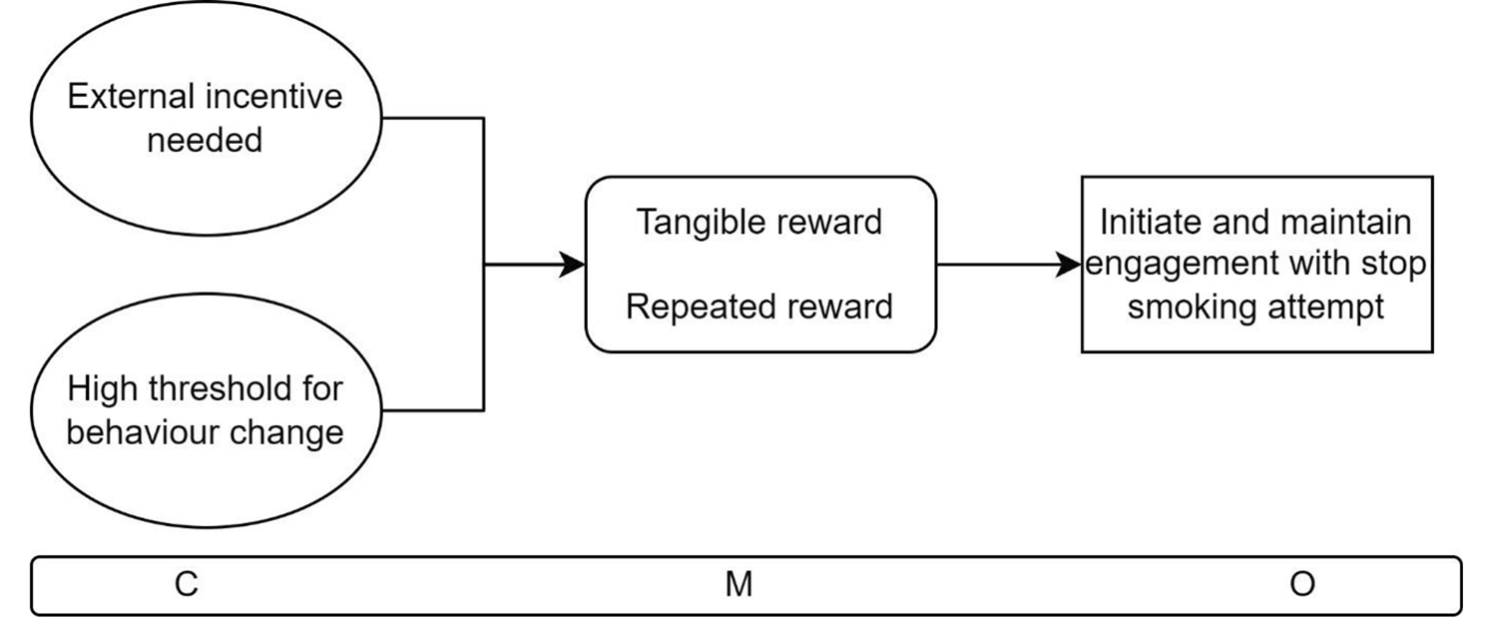
CMOC2

The second CMOC (Fig. 5) describes two contexts which trigger similar mechanisms. When people who smoke have a high threshold for undertaking behaviour change and, relatedly, when they need tangible, external rewards to make the effort to undertake such change, a financial incentive instigates action to change their behaviour [17, 21, 22, 44–49]. This behaviour change is triggered by the mechanisms of experiencing tangible and repeated reinforcement [20–22, 40, 41, 43, 46]. These mechanisms prompt people to make a stop smoking attempt and reinforce the new behaviour pattern.

### CMOC3

When a woman who smokes becomes pregnant and she is offered a financial incentive to stop smoking (C), the financial incentive can promote positive feelings for the woman because she is supported in engaging in a healthy habit for her unborn child and she is given the opportunity to provide extra financial resources for her family and/or be able to treat herself(M). The outcome is that the woman is more likely to engage with and maintain ongoing engagement with stop smoking services and have a successful stop smoking attempt (O).

**Fig. 6:**
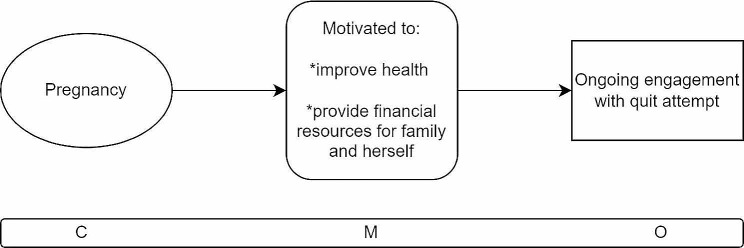
CMOC3

The third CMOC (Fig. 6) describes what happens when a woman who smokes becomes pregnant or has recently had a baby. In this context, when offered a financial incentive to stop smoking, several mechanisms are triggered for the woman in question. The financial incentive supports an existing desire many pregnant women have to embrace healthy behaviours such as stopping tobacco use, for her own and her child(ren)’s health [22, 35, 37, 39, 41, 46, 50]. It provides an extra push for the woman to attempt to give up smoking. Further, the financial incentive offers the woman an opportunity to provide financially for her growing family and/or to be able to treat herself while pregnant. For example, women reported that using the incentive money to buy utilitarian items like nappies for their baby or maternity clothes for themselves was encouraging as it supported their family’s needs. Other women described that being able to buy a nice bubble bath or bath salts was a positive experience because it allowed them to mind their own wellbeing with the money provided [22, 35, 39].

### CMOC4

For people who smoke and come from a deprived background it is common to internalise societal messages which blame individual behaviour for poor health outcomes which are, at least in part, caused by structural conditions such as poverty (C). In such a context, financial incentives for giving up smoking act as a reward and promotes feelings of autonomy, confidence, self-esteem, and respect in people who otherwise do not often feel valued or seen (M). As a result they are more likely to make a stop smoking attempt and to keep engaging with services (O).

**Fig. 7:**
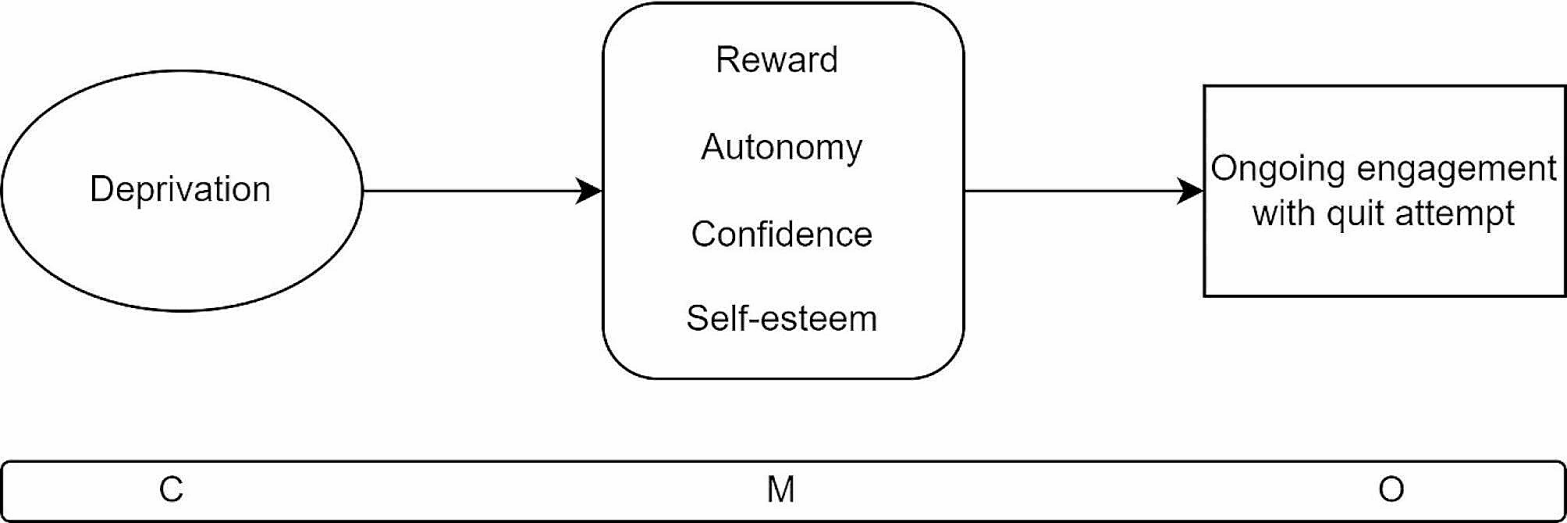
CMOC4

The fourth CMOC (Fig. 7) describes the context in which people who come from areas that experience significant deprivation, beyond financial insecurity as described in CMOC1, are on the receiving end of ongoing negative messaging about people like themselves, which they sometimes internalise and which can cause low self-esteem. With such experiences, people can feel that they are being judged by others in society for their health behaviours and choices [22, 35, 40, 41]. In such circumstances, financial incentives have the potential to trigger the mechanism of providing transformative, positive experiences where people are acknowledged for doing something well in part due to the payment but also due to the frequent engagement with counsellors who are often encouraging and during regular meetings for ongoing nicotine monitoring. Mechanisms that can be triggered include feelings of self-esteem, confidence, autonomy and being valued and seen. Financial incentives can be experienced as a reward for a job well done [21, 22, 35, 37, 39, 41, 44]. The outcome is that people undertake a stop smoking attempt and maintain their engagement with services because it offers such a positive experience.

### CMOC5

Where a social network and peer group normalises smoking (C), a financial incentive legitimises a quit attempt, providing cover for other reasons which may be less accepted within the peer group (M) and as a result a quit attempt is more likely (O).

**Fig. 8:**
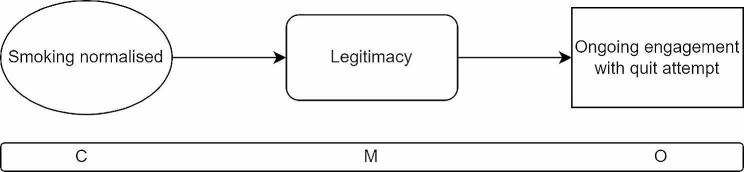
CMOC5

The fifth CMOC (Fig. 8) explores what happens when a person who smokes would like to quit but is connected to a social network where smoking is common and socially acceptable. In these cases, a person may be encouraged to keep smoking to not be seen to challenge social norms and might not feel supported in a quit attempt [40, 41, 46, 47, 51]. For such people, a financial incentive to stop smoking provides an excuse or ‘argumentative cover’ to help explain why they are no longer smoking. They can head off any questions from their peer group by referring to the financial incentive which can lend legitimacy to their stop smoking attempt. In such cases, an internal motivation present in the person seeking to quit may be present at the outset and the incentive triggers a mechanism of providing cover and allowing the person to undertake a desired quit attempt in a socially acceptable manner [39, 46, 47, 50]. The outcome is that the person is able to undertake a quit attempt without alienating their friends and family.

### CMOC6

Where service users are not motivated by financial incentives or where they find the requirements needed to receive the incentive too cumbersome, not worth the effort, or in conflict with other life tasks and commitments (C), participants may feel unmotivated or may worry how they will be perceived if they fall short of quitting (M) and as a result may disengage from the programme (O).

**Fig. 9:**
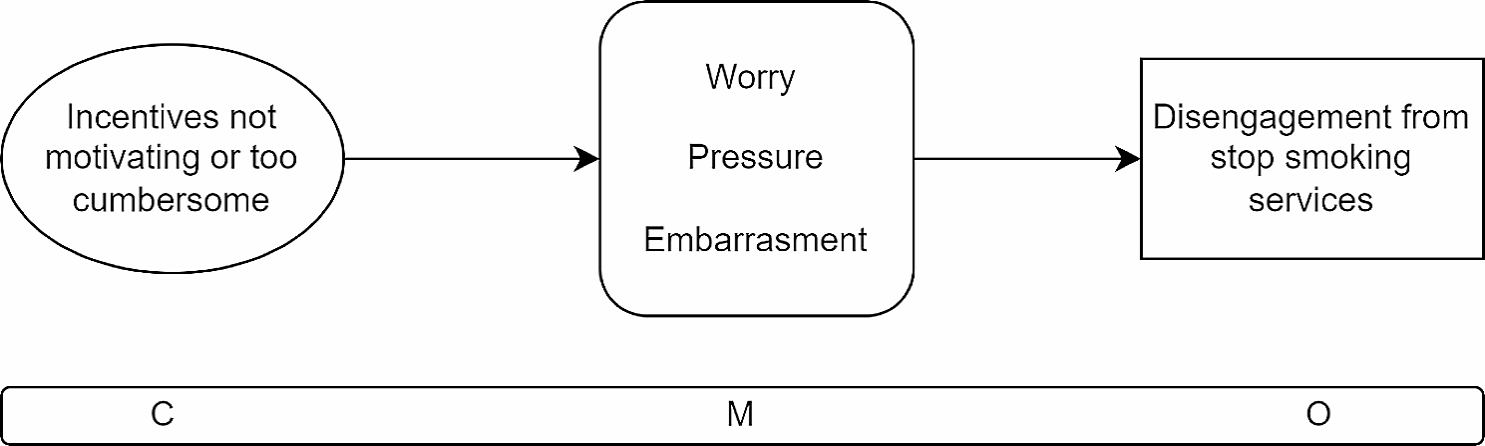
CMOC6

The sixth CMOC (Fig. 9) addresses situations where financial incentives do not work to motivate people who smoke to give up and instead act as barriers to participation. In this context, financial incentives elicit a negative response, making people feel pressure to engage with services when perhaps they did not feel they were ready or were unable to dedicate the needed time and attention to a quit attempt [35, 41, 52]. Additionally, a person undertaking a smoking attempt who has a relapse may find that the presence of a financial incentive causes them to feel worried and embarrassed about letting down the programme or service they are accessing [35, 52]. Finally, for some people the significant engagement required with stop smoking services when a financial incentive is provided in exchange for engaging with ongoing monitoring and verification of non-smoking status, such as regular exhaled carbon monoxide testing, triggers feelings of obligation and can feel like too much of an effort which takes time away from other responsibilities [22, 39, 44, 52]. As a consequence, people will not want to engage with stop smoking services and may be better served by a more traditional stop smoking programme which does not include financial incentives.

### Overarching programme theory

Putting all the findings together, our overarching programme theory (Fig. 8) combines the 6 CMOCs explored above and provides a temporal perspective on the relationship between them all. Specifically, we show that some mechanisms are likely to be triggered early in a stop smoking attempt, such as those explored in CMOC2, when a person needs external, tangible rewards to initiate behaviour change. Meanwhile other mechanisms are triggered once a quit attempt is ongoing where it sustains the person’s motivation.

Ormston et al. say of financial incentives that early in the process, they operate as an encouraging ‘tipping point’ which gives an extra boost for someone stopping smoking [21]. Later in a stop smoking attempt, they say, the incentive acts as something to look forward to which provides recognition of the effort put in and the resulting achievements, promoting well-being and self-esteem:

the incentive was viewed as: a ‘reward’, ‘bonus’ or ‘wee treat’ for quitting; a ‘wee bit extra to keep you going’ with the quit attempt; providing ‘something to work towards’; and a reason to stick with the programme or to keep going back to the pharmacist.. Similar views were expressed by service providers, who suggested that the incentive encouraged participants to stick with formal support for longer, which in turn increased their chances of quitting successfully [21].

The presence of an incentive early in a quit attempt seems to get more people to take the first step as Breen et al. explain:

‘Some participants expressed that vouchers were an incentive for behaviour initiation. For example, one participant explained that initially the vouchers were a great motivator, until the pleasure of not smoking maintained their desire to stay abstinent’ [44].

Similarly Notley et al. report that:

‘With regard to smoking cessation, where individuals may initially find quitting difficult but may adapt over time to this change, offering rewards that can initiate cessation seems to suggest that the long-term effect overall may be maintained. This is plausible, because the incentives serve to support the initial, most difficult weeks (or months) of a quit attempt and the risk of relapse reduces over time’ [17].

Other benefits will become apparent after a stop smoking attempt is under way, such as saving money from not buying tobacco products, health effects, and the ‘pleasure of not smoking’ [44] as highlighted above. Ormston et al. also report that CO testing itself was viewed as useful ‘in demonstrating immediate health benefits from quitting’: ‘I mean it made me feel good that. I kind of had pictures in my head of my lungs getting better’ [21].

Other mechanisms seem to be triggered throughout a stop smoking attempt. The value of money and the role it can play in someone’s life, giving opportunities for making autonomous choices which may not otherwise be available, is important at the beginning and throughout a stop smoking attempt. It presents the option for people to buy things which they were otherwise not able to afford and is a strong motivator especially for people on a low income or from communities with a high level of deprivation [20, 35, 37, 39, 42].

Crossland et al. remark:

Cash and shopping vouchers can function as both hedonic and utilitarian incentives. The ‘immediate and fun’ nature of shopping vouchers was considered important to compensate for the perceived loss of enjoyment arising from behaviour change e what people would be ‘prepared to get in return for not smoking’. They were seen as a ‘reward’ which enhanced feelings of wellbeing: ‘*I was over the moon with it. I was. I was really happy with it and just receiving my wee £100 one there, I was really quite chuffed’* (33, I, pregnant woman) [39].

Radley et al. reported a quote from a study participant who was on benefits and was given extra money for groceries:

‘I did want to stop smoking and although I wasn’t really a heavy smoker I felt, well I could really do with the £12.50 a week for ASDA [supermarket] tokens that would really be good for me, because I was on benefits’ [35].

Meanwhile, Breen et al. reported that a participant in their study explained that the vouchers they got in exchange for stopping smoking had allowed them to buy a television [44].

A number of studies discussed the opportunity financial incentives provided for study participants to enjoy themselves and boost their wellbeing because of the extras they could afford with the financial incentive [21, 37, 39, 41, 42, 50]. Crossland et al. reported:

‘the appeal of this incentive category seems to be their hedonic value, due to their potential to enhance a woman’s emotional wellbeing.. which it turn could increase her capacity to cope with the challenges of new behaviours’ [39].

Van den Brand et al. similarly say:

Most participants liked and appreciated the vouchers as a reward for success in quitting. ‘I like it; I’ve been using it to do fun things, too’ (participant 17, successful quitter, moderate income) [37].

As a quit attempt gets under way, people experience additional mechanisms that supplement or replace earlier mechanisms. Over time, financial incentives come to promote confidence as the effort people are making is continually and reliably rewarded. In this vein, Crossland et al. report:

Women felt that the effort they put into behaviour change deserved recognition and validation: ‘Yeah, I think it has to be for you because you’re the one that’s doing it, no one else is’. One woman who had been part of an incentive programme for breastfeeding remarked on the ‘really well thought out nice gifts’. It appears that this participant inferred some thoughtful deliberation behind the choice of incentives used, and consequently according them significance beyond their financial value [39].

Incentives help people undertaking a quit attempt feel valued and allow them to build increasing self-esteem and self-efficacy [21, 22, 39, 41, 44–46]. Thomson et al report that women in their study felt valued, more confident and privileged as a result of the financial incentive:

consumers and professionals in our study reported that unrestricted vouchers can promote individual autonomy for the most disadvantaged through providing a rare opportunity for choice and self-reward.. [and] that incentives would provide vulnerable individuals with one of the first opportunities to receive a reward and acknowledgement for an achievement’ [41].

Because financial incentives are repeated over time, contingent on behaviour change, they play a role to ensure continued participation in stop smoking programming and activities which lead to a higher likelihood that a stop smoking attempt is successful [20–22, 35, 41, 44, 46, 51]. In this way, the incentives indirectly facilitate higher engagement with services than can be the case in other stop smoking programmes, according to Mantzari et al.:

The effectiveness of financial incentive schemes in changing behaviour might also result from indirect influences, mediated by changes to some aspects of the process involved in their delivery. For example, the provision of incentives requires contact between health professionals, who measure achievement of the target behaviour, and patients. Incentives might therefore operate by increasing health professionals’ engagement with patients or through the additional involvement required on behalf of the latter, such as attending clinics or undergoing particular tests, as part of assessing eligibility for a reward [22].

As a result, participants in a stop smoking attempt may experience a sense of responsibility through the frequent contact with health professionals or stop smoking counsellors [21, 22, 35, 44, 52]. Breen et al. reports: ‘one participant who joined the program with his wife expressed how the program had made them feel accountable for committing to give up’ [44]. Frequent contact also provides frequent opportunities for experiencing success according to Ormston et al.:

‘Participants’ accounts suggested that the CO test may have helped provide a focus for encouragement, praise and support. “She made it good to go in there and you know, breath into your wee machine… And she got just as excited as you when it was just on the little ‘1’ thing.”’ [21].

Additionally, Mantzari et al. report that the biochemical tools for measuring adherence were themselves motivating:

witnessing improved carbon monoxide levels and/or receiving related praise from the smoking cessation counsellors was perceived to increase confidence and was thus perceived as facilitating efforts: “It’s just more of a moral support I think really and checking your carbon levels and once you realise you’ve done good, you know, it boosts your confidence to keep, keep not smoking, do you know what I mean?” [22].

Conversely, however, as discussed above in CMOC6, financial incentives and their delivery can also make people feel unwelcome pressure to meet the required behaviour change as reflected in several of the included articles. One article in particular by Allan et al. [52], reports on data collected via qualitative interviews with representatives of the roughly 35% of participants in the Scottish quit4u programme who registered for the programme but did not engage with it. These interviews were directly focused on the negative aspects of the quit4u intervention using financial incentives. The study found that interviewees viewed the contractual nature of the financial incentive negatively:

Reasons for disengagement hinted at a felt change in clients’ relationship with service providers, with the incentive introducing a quasi-contractual relationship. This placed the patient in the role of “providing the service” (smoking cessation) and the health care professional as the “buyer”. For some, this relationship manifested itself in a sense of obligation to the service providers, manifest most clearly when they had “failed to deliver”:

“..I just felt I had let them down as well. Even though it is yourself, you still feel as if you are letting other people down as well which again is a horrible feeling so then you feel guilt again and I think, I’m just gonna have a fag.” (female, 35, lab worker, group 3) [52].

Allan et al. also reported that the effort to participate, such as the time required to travel to the locations where carbon monoxide testing took place and the particular times when it was available, was too great to be worth the incentive for some of the people interviewed. Unlike participants in a number of the other studies, who viewed the ongoing interaction with stop smoking services as a facilitating factor, participants in this study found the sense of obligation to present unmotivating pressure and lead to worry about letting the programme down. Altogether, these negative experiences and feelings led to disengagement from a stop smoking attempt and ultimately to a lack of success.

**Fig. 10:**
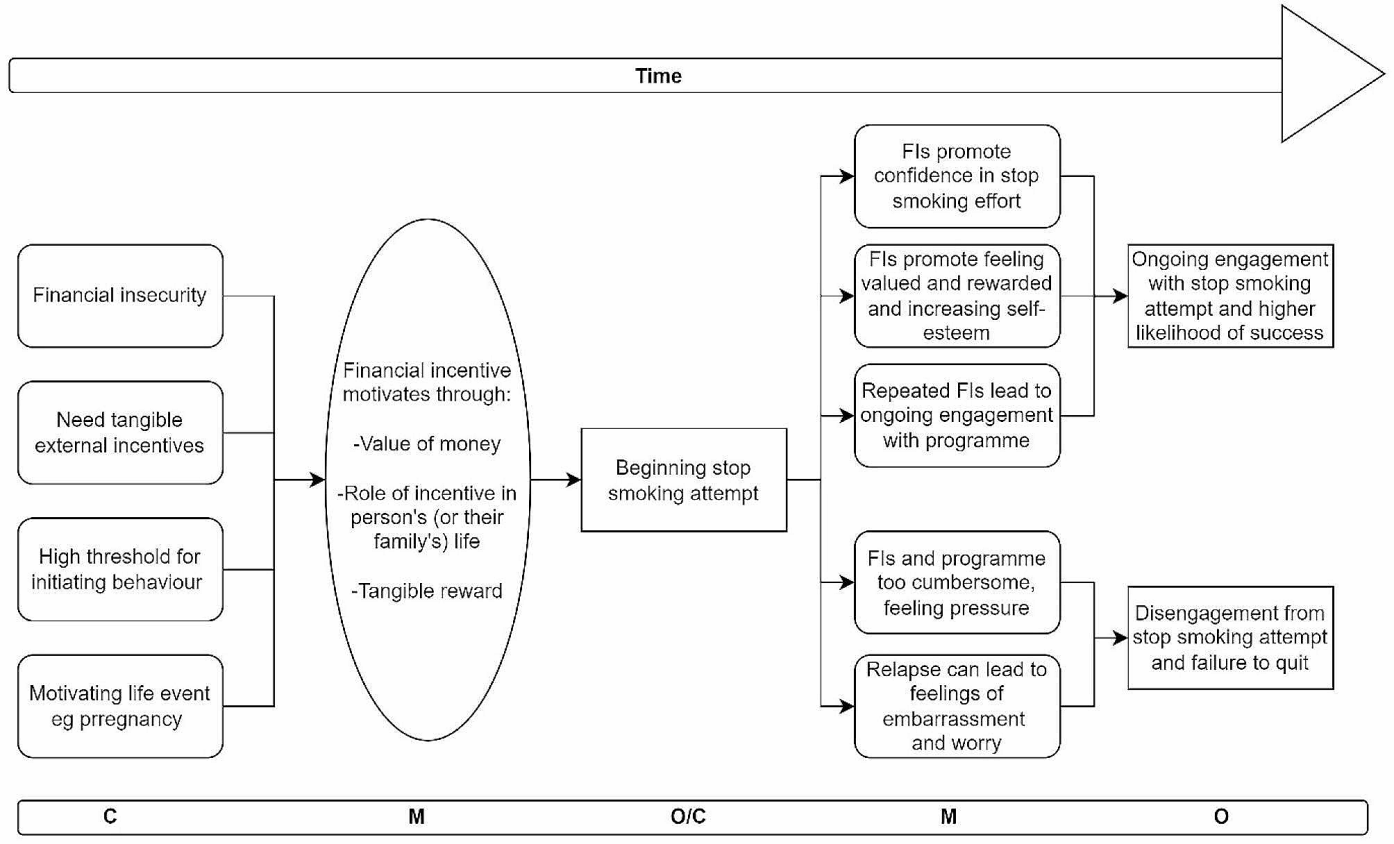
Overarching programme theory

Putting all these findings together and consolidating the six CMOCs outlined above, Fig. 10 presents the overarching programme theory for this realist review. This graphic representation of the complex relationship between financial incentives and stopping smoking occurring over a period of time is necessarily more simplistic and linear than the actual process of stopping smoking which is notoriously difficult for most people who smoke. In any stop smoking journey whether in using no supports, community supports, or in a structured programme, a person will often try to stop several times using different modes of support which means that they will often enter and exit the stop smoking process repeatedly, potentially at different points along the programme theory due to changing contexts and with a variety of mechanisms triggered dependent on what is going on in their life [53–55].

## Discussion

Given the continuing scale of harm caused by smoking, effective implementation of innovation in stop smoking care is urgently required [6], especially in population groups which risk being left behind [16]. The efficacy of financial incentives in helping people to stop smoking has been demonstrated in other literature, such as in the 2019 Cochrane review on the topic, and more recent studies [17, 44, 49, 56–61]. Trials which offer financial incentives see higher stop smoking rates among the intervention group compared with controls.

While there are increasing calls for more widespread implementation of financial incentives in practice, evidence of why this complex intervention works and whether it works in different ways for different people is needed to translate evidence of efficacy into programmes which are effective in the real world [19, 62]. Our realist review presents causal pathways in this complex topic where health interacts with social factors, gender, income, class, and other aspects. Without the kind of evidence presented here, policy makers and programme designers seeking to design and implement a complex stop smoking intervention using financial incentives may struggle to know how to design it, who to target, how to deliver it, and how to monitor and evaluate it for effectiveness and unforeseen consequences. Implementing a complex intervention in an existing ecosystem of health interventions is notoriously difficult because they often need significant and stable funding, staffing, resourcing and training, and have to be placed in existing structures with their own priorities and agendas [63].

We synthesised 22 studies into an overarching programme theory which explains that financial incentives work in different ways for different people across time. At the start, they work when a person’s interest in stopping smoking is sparked or reinforced by the offer of an incentive by encouraging the reluctant and those who need extra incentives to take up new and difficult behaviour patterns. Later when a person is well engaged with a stop smoking effort, financial incentives work through reinforcing the importance and value of the stop smoking attempt and the person undertaking it, through ongoing verification of abstinence leading to accountability, and through building confidence and self-esteem via encouragement and rewards.

Our findings suggest that stop smoking programmes providing financial incentives should understand what motivates a given client and the potential barriers they may face. Framing financial incentives to support the innate motivations experienced by a person seeking to stop smoking, such as positive peer pressure or health motivations due to a change in their life such as pregnancy, or, to help them overcome a specific barrier, such as having a high threshold for initiating a new behaviour, will make the incentive more meaningful for the individual. Additionally, if a stop smoking counsellor is able to link the financial incentive to needs in the person’s life and the ability to create financial opportunities which would otherwise be off limits, they offer a powerful opportunity for triggering mechanisms within individuals that motivate them to begin and maintain a stop smoking attempt.

Stop smoking counsellors should be aware that receiving financial incentives can increase confidence and self-esteem and recipients can feel valued and rewarded. Supporting and enhancing those feelings may lead to a stop smoking attempt being potentially a powerful and transformative experience, as reported in several of the included articles [17, 22, 39, 41, 45].

Several data sources suggested that some pregnant women were very focused on their unborn child when undertaking a stop smoking attempt while others suggested that a pregnant woman can feel that their individual identity and needs are somewhat overlooked when attention is focused entirely on her growing baby [22, 35, 39]. In the prior instance, providing an opportunity to gain financial resources and to reduce health risks for her baby is encouraging for the client. In the latter instance, she may be encouraged by the opportunity to engage in self-care practices after using the financial incentive.

It is likely that many pregnant women will experience both a desire to look after their baby and feel that their own wants and needs can get lost in the pregnancy experience. For such women, the relevance of a financial incentive to her life is important. Some women reported that they enjoyed vouchers which could be traded for pamper gifts, while other women felt that vouchers should be for baby items only. A successful intervention will provide flexibility to meet the particular needs and desires of a particular woman at a given time in her pregnancy [39, 41]. Cash incentives allow the person stopping smoking to meet their exact needs where vouchers are less flexible [42].

Findings exploring how financial incentives can fail to motivate a person to stop smoking, give clues for pitfalls which stop smoking interventions and staff can seek to avoid. It is likely that the high level of interaction with services required when receiving a financial incentive in exchange for bio-verified abstinence will not suit all people at all stages in their lives. Stop smoking counsellors can seek to identify whether a person has capacity for it at a given time. CMOC6 also suggests that people who experience a relapse can worry about how it will be perceived by stop smoking counsellors. It is important that such moments, which are very common in a general stop smoking journey which may include attempts at stopping smoking by oneself as well as engaging with several organised programmes, are met with empathy and encouragement with the ultimate goal of getting the person through a successful stop smoking attempt in the future. For that reason, stop smoking programmes should ideally be provided by people who understand the difficulties of tobacco addiction and are familiar with the typical course of quitting, including relapse, so rapport can be built and counselling can be provided in a person-centred way without pressure.

### Limitations

This review has several limitations. First, like any review it depends on the published literature and as noted throughout this article while there is sufficient evidence to suggest that financial incentives work to help people stop smoking there is still a need for more evidence of how, why, for whom and to what extent this is the case. As the review builds on the evidence currently available, it may possibly have missed important contexts and mechanisms which have not yet been described. Second, the review only included studies in English.

## Conclusion

This is the first realist review of how financial incentives work to help people stop smoking. The need for more ‘real-world’ evidence to support policy and practice in stop smoking care is well-recognised [62]. This study demonstrates the role realist approaches can play in complementing traditional studies of intervention efficacy by deepening the evidence base needed to support implementation of complex and emerging innovations in stop smoking care, especially for population groups which risk being left behind in existing stop smoking services. It is timely given that implementation of financial incentives in stop smoking services is now becoming mainstreamed in some countries [64].

We have presented findings which will assist tobacco control policy makers and stop smoking care practitioners in decisions on designing and implementing stop smoking interventions using financial incentives or adding financial incentives to existing programmes. Our synthesis indicates the kinds of experiences and conditions in which a person who smokes might be particularly motivated by an offer of financial incentives. Understanding for whom incentives work, and work differently over time, and why, is key.

### Electronic supplementary material

Below is the link to the electronic supplementary material.

Supplementary Material 1

Supplementary Material 2

## Data Availability

Data used for the construction of CMOCs 1–6 can be found in the supplementary file.

## Abbreviations

CMOC: Context-Mechanism-Outcome configuration
