# Supplementary material for: How and why do financial incentives contribute to helping people stop smoking? A realist review
Source: BMC Public Health. 2024 Feb 16;24:500. doi: 10.1186/s12889-024-17967-3 (PMC10873947; doi:10.1186/s12889-024-17967-3)
Supplement: Supplementary file 1 — Supplementary Material 1 [file 12889_2024_17967_MOESM1_ESM.docx]

Supplementary file 1.pdf

1. **Search terms**

We searched five databases on 16 February 2022: Medline ovid, Embase.com, Scopus, PSYCHInfo and CINAHL.

The search terms were as follows, with modifications to suit the parameters of each database:

Smoking cessation (MeSH type term) OR Smoking cessation (keyword) OR stop* N3 smok* OR quit* N3 smok*) AND (financial N3 incentive* OR economic N3 incentive* OR money N3 incentive* OR monetary N3 incentive* OR cash N3 incentive* OR pay* N3 incentive* OR award* OR token* OR prize* OR voucher*

The selection of databases was updated between the publication of our Prospero registration (<https://www.crd.york.ac.uk/prospero/display_record.php?RecordID=298941>) and the systematic search after consultation with a subject librarian. He suggested including PSYCHInfo and leave out the Cochrane Library as any articles published there would be in the other included databases. He also informed us that OpenGrey is no longer being updated and therefore suggested to leave it out of the systematic search. Our study protocol reflects the updated search strategy.

1. **Glossary of terms**

Context: environments, settings, circumstances or structures that trigger mechanisms. Anything which triggers, impedes or blocks the action of a mechanism[1]

Context-mechanism-outcome configuration (CMOC): configuration that explains the causative relationship between a mechanism which is triggered in a given context and as a result produces an outcome[1]

Mechanism: a pre-existing, latent causal power or force (eg norms, belief systems, gender, class and sequential processes) which is activated in a particular context leading to an outcome. Mechanisms cannot be directly measured or seen[1]

Outcome: impact, change or action arising when a particular mechanism is activated in a particular context[1]

Programme theory: a combined set of theoretical explanations of how a particular process, intervention or programme is expected to work[1]

Realist approach: the realist approach to research used in realist review and realist evaluation is a theory driven way to explain generative causation in areas of study that are highly complex and in which empirical testing is not possible[1]

Relevance: the determination of whether a particular study, report, article etc is relevant to the research question[1]

Rigour: the determination of whether a particular finding or piece of information in a given source was arrived upon in a way that was robust and faithful to the particular method being used. If a study was done well adhering to its method it is more likely to be rigorous however all sources of data can yield pieces of helpful information[1]

1. **Supporting data for CMOCs**

### CMOC1

###
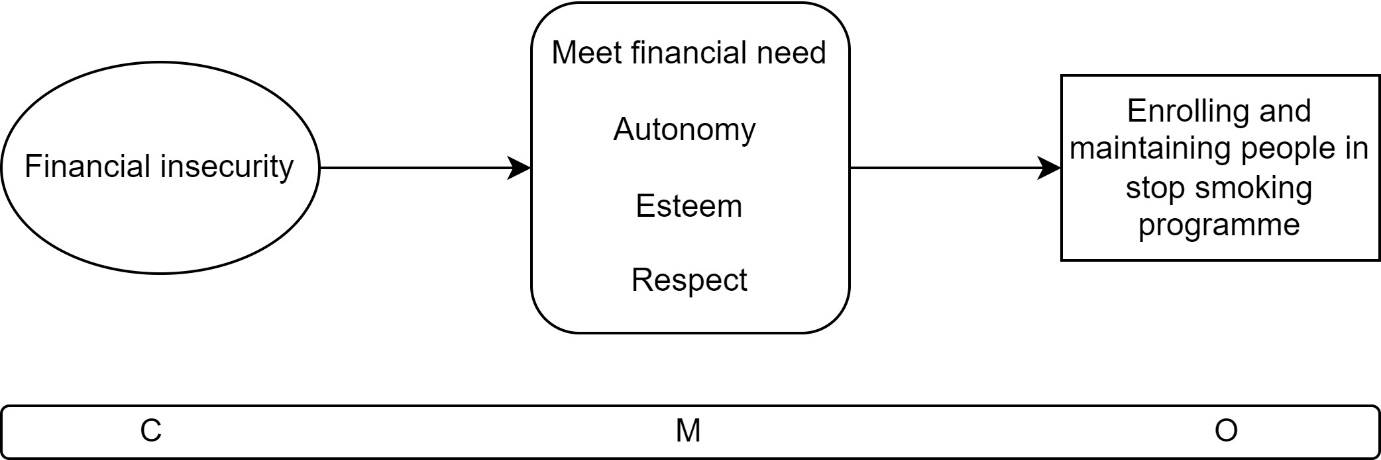


When a person who smokes also experiences financial insecurity (C), a financial incentive encourages them to initiate a stop smoking attempt and to engage with the wider stop smoking programme because it offers an opportunity to meet financial needs, gives a sense of autonomy due to having extra financial resources, and engenders feelings of trust and respect (M). This leads to more success in enrolling and maintaining people in stop smoking programmes (O).

Alternative positive and negative reinforcers are commonly present in smoker’s naturalistic environment and are known to inﬂuence smoking behavior. Financial concerns are among most common reasons given by smokers who attempt quitting (McCaul et al., 2006) and ﬁnancial incentives for not smoking can induce abstinence in treatment-seekers. (Bisaga et al., 2007)

An interesting finding was that the few participants who mentioned that the incentives were motivating had a lower income. It is likely that financial incentives are valued more by smokers who have less money to spend. Although previous research has not yet found a decisive answer on this hypothesis, a systematic review found evidence that financial incentives may be more effective in influencing health behaviour in participants with a higher deprivation level. (Van den Brand et al., 2018)

One subject thought that the voucher could subconsciously have contributed to quit success. ‘Subconsciously, it will indeed be a stimulus that makes you think: Oh, that's a nice added benefit’ (participant 3, successful quitter, low income). Another participant thought that the voucher had been a motivator to stay abstinent from smoking. ‘Yes, it did factor in. I think that it did actually serve as a motivation to keep going’ (participant 2, smoker, low income). (Van den Brand et al., 2018)

Participants were asked how they felt about the incentive. Most participants liked and appreciated the vouchers as a reward for success in quitting. ‘I like it; I’ve been using it to do fun things, too’ (participant 17, successful quitter, moderate income). (Van den Brand et al., 2018)

Reasons to Enroll in Incentive Programs

The most common reason participants would consider enrolling was because the money would affect the individual’s life (43.54%), followed by the money compensating how hard it would be to quit (32.65%), and being paid signaling the importance of quitting (14.97%). (Breen, Ferguson, et al., 2021)

Other participants considered the FIs a reward for their success; for example, one participant explained how the vouchers had allowed them to buy a television as their reward for abstaining from smoking. (Breen, Frandsen, et al., 2021)

Some participants expressed that vouchers were an incentive for behaviour initiation. For example, one participant explained that initially the vouchers were a great motivator, until the pleasure of not smoking maintained their desire to stay abstinent. (Breen, Frandsen, et al., 2021)

The greater the perceived relevance or personal value of an incentive, the more appealing it will be to that individual. Hence individuals may value vouchers at less than their equivalent monetary worth, meaning voucher-based FIs may need to be of a greater value than cash-based FIs to ensure the same utility is afforded some research. In support of these the indicates vouchers are valued at only 80%–90% of their monetary amount. (Breen et al., 2022)

The most consistent observation across the present and prior studies is that participants rated the challenge of abstaining from smoking as progressively easier as the duration of abstinence increased. As noted previously (Lussier et al.), lower response effort has been demonstrated in basic and applied research to increase the probability of responding (Friman & Poling, 1995). To the extent that abstaining from smoking can be considered an operant response or task (which we believe is clearly supported by the present results), a history of successfully abstaining coupled with a progressive increase in the perceived ease of abstaining could reasonably be expected to be associated with an increase in the probability of sustaining abstinence in the future. In population surveys of current smokers, perceived difficulty associated with quitting appears to deter smokers from attempting to quit (Mullins & Borland, 1996). (Chivers et al., 2008)

Cash and shopping vouchers can function as both hedonic and utilitarian incentives. The ‘immediate and fun’ nature of shopping vouchers was considered important to compensate for the perceived loss of enjoyment arising from behaviour change e what people would be ‘prepared to get in return for not smoking’. They were seen as a ‘reward’ which enhanced feelings of wellbeing: I was over the moon with it. I was. I was really happy with it and just receiving my wee £100 one there, I was really quite chuffed (33, I, pregnant woman). (Crossland et al., 2015)

Our ﬁndings suggest that incentives may be more effective if they offer autonomy, pleasure and convey esteem. (Crossland et al., 2015)

While those enrolling did not differ from those not enrolling in terms of social deprivation, those enrolled who made a quit attempt were less deprived. Providing additional support to those who enrol but do not initiate a quit attempt may prove effective in increasing those initiating a quit attempt, as well as reducing the social gradient in those initiating and sustaining a quit attempt. (Ierfino et al., 2015)

However, the reinforcing efficacy of money may still depend on relatively chronic states of motivation (eg, limited access to food or other resources due to poverty). Therefore, the value of monetary incentives may be enhanced among individuals who are severely economically disadvantaged, as has been suggested within the context of some CCT programs. (Meredith et al., 2014)

With regard to smoking cessation, where individuals may initially find quitting diﬀicult but may adapt over time to this change, oﬀering rewards that can initiate cessation seems to suggest that the long-term eﬀect overall may be maintained. This is plausible, because the incentives serve to support the initial, most diﬀicult weeks (or months) of a quit attempt and the risk of relapse reduces over time. Findings from our meta-analysis in mixed populations suggest that incentives continue to have a significant impact on sustained smoking cessation, even aﬅer they have finished. (Notley et al., 2019)

[participant quote:] What was important to me was the money really. Cos it could have done so much. With being pregnant and struggling on Job Seekers Allowance with nae top up (.. .) The money was the thing that actually edged me towards it (the scheme). (Radley et al., 2013)

[participant quote:] I did want to stop smoking and although I wasn’t really a heavy smoker I felt, well I could really do with the £12.50 a week for ASDA tokens that would really be good for me, because I was on benefits. (Radley et al., 2013)

. . consumers and professionals in our study reported that unrestricted vouchers can promote individual autonomy for the most disadvantaged through providing a rare opportunity for choice and self-reward. Similarly discourses of incentives as ‘unfair’ for rewarding ‘bad behaviour’, of discouraging individual responsibility or for targeting only disadvantaged communities contrast with narratives of feeling valued, more confident and improved self-esteem. Our interpretation is that media debates on the advantages and disadvantages of financial incentives can be easily biased towards intellectual philosophical, political and ethical arguments about the role of the state, without considering the perspectives of more disadvantaged families who are struggling to do the best for their children. This is particularly important as children have no choice and public acceptability is greatest for incentives to protect their wellbeing [2] (Thomson et al., 2014)

A few professionals reflected that incentives would provide vulnerable individuals with one of the first opportunities to receive a reward and acknowledgement for an achievement. Unrestrictive incentives like shopping vouchers could provide the most disadvantaged families with a rare opportunity for autonomy to ‘make decisions about what they ought to be spending the additional money on’, such as providing ‘treats’ for themselves and their families. (Thomson et al., 2014)

Among those who felt it had been a factor in encouraging them to stay quit, the incentive was viewed as: a ‘reward’, ‘bonus’ or ‘wee treat’ for quitting; a ‘wee bit extra to keep you going’ with the quit attempt; providing ‘something to work towards’; and a reason to stick with the programme or to keep going back to the pharmacist. ‘I was going to stop anyways. (It was) just a, certainly a bonus having that there like. I reckon it would probably encourage you to keep going back to the chemist.’ (P209, quit) Similar views were expressed by service providers, who suggested that the incentive encouraged participants to stick with formal support for longer, which in turn increased their chances of quitting. (Ormston et al., 2015)

### CMOC2

###
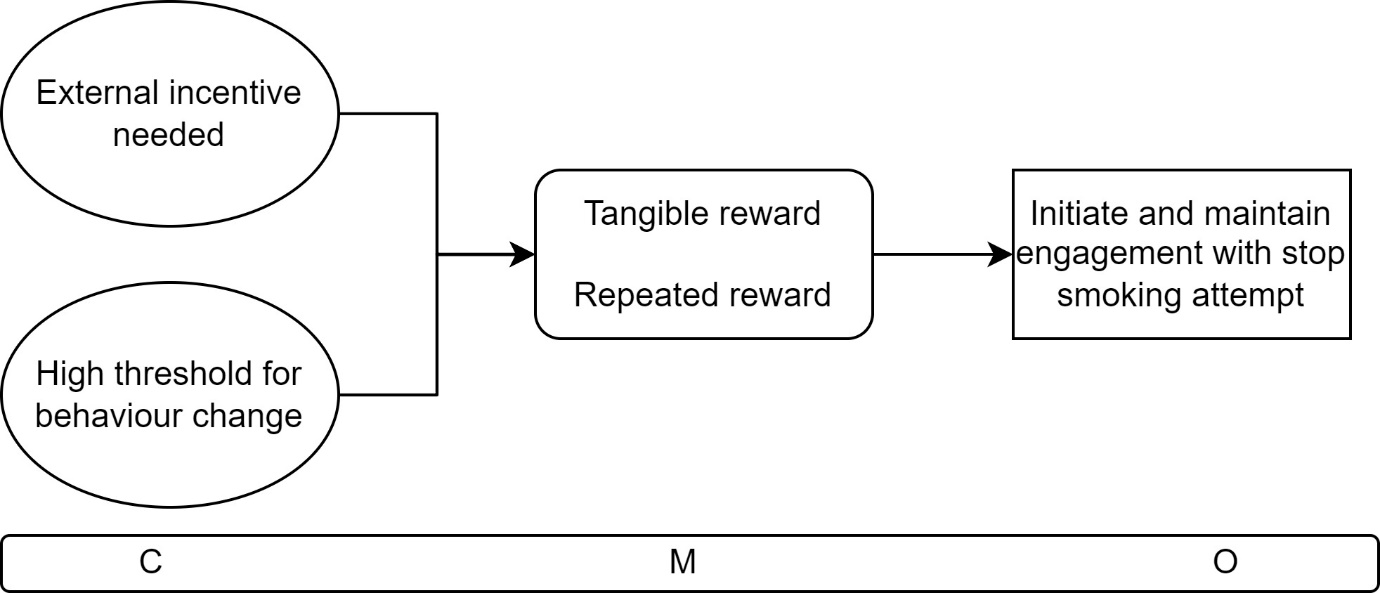


When people who currently smoke have a high threshold for behaviour change and need tangible and external rewards to initiate change (C), a financial incentive encourages action through the presence of repeated external reinforcement via a payment and offers an opportunity for utilitarian and/or enjoyable spending (M) to initiate and maintain a stop smoking attempt (O).

A potential explanation of the association between the incentives and self-efficacy is that the incentives encourage people to visualize achieving their goal of quitting smoking and receiving the reward. This visualization of successfully performing a behavior (such as quitting smoking) can enhance a person’s self-efficacy for that behavior. Because a high self-efficacy has shown to be a good predictor of successful behavioral change, financial incentives may be a novel way to increase

self-efficacy and improve quit success which should be explored in further research. (van den Brand et al., 2021)

Some participants expressed that vouchers were an incentive for behaviour initiation. For example, one participant explained that initially the vouchers were a great motivator, until the pleasure of not smoking maintained their desire to stay abstinent. (Breen, Frandsen, et al., 2021)

That is encouraging in that it suggests that regular exposure to smoking paraphernalia or brief smoking lapses need not scuttle a cessation effort when there are strong incentives to sustain abstinence, as there were with the contingency management intervention employed in the current study. Many of the difficult-to-treat populations with whom contingency management procedures for smoking cessation hold promise reside with other cigarette smokers, which makes exposure to secondhand smoke and the probability of relapse more likely (Solomon et al., 2007). The present results suggest that at least for the period while the reinforcement contingencies are in place, abstinence may still be sustainable in those populations and settings. (Chivers et al., 2008)

There are sound scientiﬁc rationales for the systematic use of ﬁnancial incentives to treat addictions. Most fundamentally, the approach is based on well-established principles of operant conditioning, that is, the study of how environmental consequences alter the future probability of voluntary behavior (Bouton, 2007). Behavior that is followed by reinforcing consequences increases in future probability while behavior that is followed by punishing consequences decreases. (Higgins et al., 2012)

Also relevant is an emerging area of behavioral economic research on delay discounting documenting that individuals with addictions discount the value of temporally delayed reinforcement more than do matched controls without SUDs (Bickel et al., 2007). That is, consequences that are delayed in time have less effect on current behavior than do more immediate consequences. The shape of the function relating delay to reinforcement value is hyperbolic, meaning that value diminishes precipitously with relatively brief delays and then levels off as delays continue to increase. This is true for humans generally as well as many other species, but individuals who have SUDs appear to be particularly sensitive to temporal delays (Bickel et al., 2007). Considering that most of the naturalistic reinforcers for discontinuing drug use (e.g., improved health of self and baby) are delayed in time while those derived from drug use are relatively immediate (e.g., euphoria, enhanced social interaction), it is perhaps not too surprising that so many individuals with SUDs struggle in trying to discontinue drug use. Knowing about greater discounting among those with SUDs also provides a rationale for why providing relatively immediate reinforcement contingent on therapeutic progress in the form of ﬁnancial incentives might be especially helpful in bridging the temporal gap between discontinuing drug use and reaping naturalistic rewards for doing so. (Higgins et al., 2012)

It is possible that the offer of ﬁnancial incentives might have ameliorated the normally detrimental impact of women’s preference for the present, by providing immediate rewards for a behaviour with typically delayed beneﬁts. (Ierfino et al., 2015)

Smokers often have preferences against using counseling or pharmacotherapy, and may cite concerns about side effects or overestimate their likelihood of successfully quitting without assistance.44–48 Financial incentives that are sufficiently large—in combination with behavioral economic strategies—may help more smokers overcome these barriers to evidence-based therapy. (Ladapo et al., 2020)

A major unanswered question in the financial incentive literature is whether to use goaldirected incentives (incentives for use of evidence-based therapies, which are widely underutilized) or outcome-based incentives (incentives for successful achievement of an outcome, like successfully quitting) for health improvement.27,48 Most smoking cessation studies applying incentives have primarily targeted the outcome of smoking cessation. However, if incentives can be used to steer patients toward evidence-based therapies that also increase intrinsic motivation (e.g., motivational interviewing or successful use of pharmacotherapy),49,50 concerns that incentives engage extrinsic motivation at the expense of intrinsic motivation may be attenuated. The optimal design is unknown.

(Ladapo et al., 2020)

One major concern about financial incentives for smoking cessation is their long-term efficacy, with critics noting that financial incentives (extrinsic motivation) may crowd out intrinsic motivation51,52 and undermine durable smoking cessation. Others have noted, however, that levels of intrinsic motivation for activities we incentivize may already be low, leaving little motivation at risk for crowd out. (Ladapo et al., 2020)

This differential engagement with the services seems related to the offer of Financial Incentives which appears to have motivated incentivised women to attend the services: “I wouldn’t have bothered going all the way to the doctors because at the beginning of your pregnancy and that you don’t want to go out the house anyway because you’re feeling sick and you’re heavy and frumpy, and it just seems like a long way to go for nothing just to blow into a thing. With the vouchers it’s like you’re getting paid... rewarded to go there” (Participant14; incentivised group). (Mantzari et al., 2012)

Indeed, the Financial Incentives were perceived as facilitating cessation attempts: “the vouchers give me incentive to like stop smoking... So the vouchers have helped yeah because I’m thinking it’s not that worth risking.” (Participant14, incentivised group) (Mantzari et al., 2012)

It is also possible however, that the effectiveness of financial incentives in achieving behaviour change might also result from an interaction between direct influences to individuals’ motivation and self-regulation and indirect influences mediated by changes so certain aspects involved in the process of incentive delivery. (Mantzari et al., 2012)

Frequently reinforcing healthy behavior can have very different effects depending on the schedule according to which incentives are delivered. One schedule of reinforcement that has been systematically examined across several clinical studies is as an escalating schedule of reinforcement with a reset contingency. In this schedule, healthy behavior have distinguished between two types of target behavior: simple (eg, immunization) and complex (eg, drug abstinence). From a behavior analytic perspective, this distinction can be conceptualized as a difference between behavior that requires only one, or a few, discrete response(s) and behavior that requires repeated responses over an extended duration. Behavior that requires only one response (eg, visiting a clinic to learn the result of a tuberculosis skin test) can be prompted by a relatively small incentive (eg, $5).155 In contrast, a more “complex” health-related behavior that requires repeated choices over an extended duration (eg, drug abstinence) might require frequent and prolonged reinforcement to establish and maintain behavior change. (Meredith et al., 2014)

Previous reviews of incentive-based interventions for smoking cessation have expressed concerns that the eﬀect of incentives may be time-limited. This would conform to a learning theory-based explanation, that rewards are eﬀective when consistently oﬀered, but that the eﬀect of the reward may be 'extinguished' when rewards cease. With regard to smoking cessation, where individuals may initially find quitting diﬀicult but may adapt over time to this change, oﬀering rewards that can initiate cessation seems to suggest that the long-term eﬀect overall may be maintained. This is plausible, because the incentives serve to support the initial, most diﬀicult weeks (or months) of a quit attempt and the risk of relapse reduces over time. Findings from our meta-analysis in mixed populations suggest that incentives continue to have a significant impact on sustained smoking cessation, even aﬅer they have finished. In our next update, when we anticipate further evidence will be available and therefore contribute further data points, we plan to conduct an analysis comparing quit rates at last incentive point to quit rates at subsequent follow-ups where incentives are not provided; this would provide a more direct test of the lasting eﬀect of incentives. (Notley et al., 2019)

Participants who felt that the incentives had been a primary or a secondary reason for their signing-up indicated that it provided a trigger or ‘tipping point’ to give up and to give up with support. These views were echoed by community pharmacists and smoking cessation advisors. (Ormston et al., 2015)

Many participants felt that the Quit and Win study gave them the increased motivation they needed to quit. ‘I wouldn’t have quit at that time if there wasn’t a contest. And I’d probably still be smoking’. (Thomas et al., 2015)

Incentives to consumers could also operate as ‘enablers’ for ongoing contact with services:

Sometimes, when you are feeling like rubbish and your house is a mess and you think ‘‘oh I can’t be bothered with somebody else coming round now’’, you’ve got the health visitor and you’ve got you know all the other…. and you think ‘‘oh another person coming round to look at me’’, but I think the incentives definitely can well …I will get a present from this one (T9, I, mother). (Thomson et al., 2014)

Regular women–provider contact, such as depicted within the Heil et al. [39] intervention vignette and the opportunity to demonstrate ‘value’ to women through incentive delivery was believed to help create a ‘positive effect’ on the women–provider relationship, assist women-centred care and make it ‘easier for staff’ to encourage women to adopt healthy behaviours. (Thomson et al., 2014)

### CMOC3
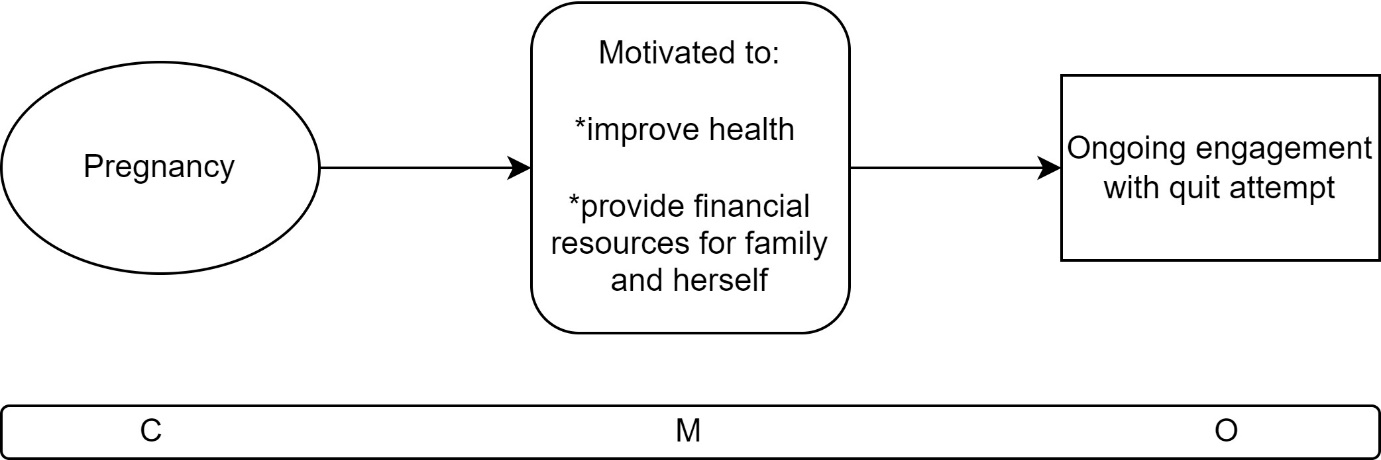


When a woman who smokes becomes pregnant and she is offered a financial incentive to stop smoking (C), the financial incentive can promote positive feelings for the woman because she is supported in engaging in a healthy habit for her unborn child and she is given the opportunity to provide extra financial resources for her family and/or be able to treat herself (M). The outcome is that the woman is more likely to engage with and maintain ongoing engagement with stop smoking services and have a successful stop smoking attempt (O).

. . . reasons for quitting smoking than the vouchers, such as their or their children’s health. (Van den Brand et al., 2018)

Women described various items they had chosen or would choose to buy with incentive vouchers or cash, such as baby items, household goods, clothing and jewellery. Unrestricted incentives such as shopping vouchers allow women to tailor the incentive to maximise their own motivation with the option to ‘save up’ for an expensive item e ‘Yeah I was thinking: pram. That's a pram’. Our interpretation is that this facilitates important strategies necessary for sustaining behaviour change such as goal setting, planning and delaying gratiﬁcation. (Crossland et al., 2015)

However, in addition to views of shopping vouchers as a ‘bonus’, some women saw shopping vouchers as potentially ‘helpful’ for people who are ‘struggling’ ﬁnancially. (Crossland et al., 2015)

Most of the tangible incentives described above have an experiential aspect, but some participants pinpointed an experience ‘like an activity event or a day with your kids or something like that’ .An experience or activity that extends beyond the individual was particularly valued. Such activities are likely to be pre-selected or allow minimal choice, are hedonic and provide opportunities for strengthening social bonds. Such experiences may be beyond the ﬁnancial and planning resources of some families, but are commonly considered an important part of being a parent and cementing family wellbeing. (Crossland et al., 2015)

‘Hedonic’ incentives were felt to increase feelings of wellbeing, thus supporting women's motivation and encouraging perseverance with the challenges of behaviour change. This is consistent with ﬁndings that maternal and family wellbeing are important drivers in women's decisions around health behaviours in the perinatal period (Hoddinott et al., 2010). Pleasure is commonly problematized in public health discourses, with pleasure seen as a key reason why individuals partake in risky or unhealthy behaviours and therefore considered an obstacle to health, while health authorities may be depicted as repressive killjoys. (Crossland et al., 2015)

The vouchers appeared to have achieved this by providing a goal to work towards and a focus for resisting urges to smoke: “I feel like I need another one [cigarette] I sort of sit there and think to myself well if I have this one it’s going to mess me up getting my vouchers for my kids.... I won’t because I’ll just think well I’ve got the vouchers to look forward to” (Participant16, incentivised group) (Mantzari et al., 2012)

Type 1: Mothers to be (see Table 6 for illustrative quotes) For this group the child and the child’s health were at the forefront of their decision-making. These were often first time mothers who had always assumed that pregnancy would be the big test; a time that they would make a serious attempt at stopping. The group also included some second time mothers who expressed feelings of guilt and wanted to “do it right” this time round. Overall motivation to quit, and in some cases, confidence, were high and quitting aids tended not to be particularly important. Some used NRT, while others expressed concerns about damage to the child. Some also introduced strict smoke free restrictions in the home and abstained from drinking as well. (Radley et al., 2013)

Because I was carrying him, it was for his sake. If I wasn’t pregnant I wouldn’t have stopped, I would have found it really hard. (Radley et al., 2013)

Participants primarily quit for family, children, and significant others. Individuals voiced a number of reasons why quitting for loved ones was important to them. These examples ranged from quitting simply because others around them did not like the habit, concern about the influence their smoking had on their children, and concern regarding the possibility of negative health outcomes from smoking on their loved ones.

‘I would say what encouraged me to quit the most was my family because they are very clear about the fact that it’s obnoxiously disgusting’.

‘My main motivator was my kids, I did not want them to have to see me die of lung cancer like my friend who watched a friend die’. (Thomas et al., 2015)

Financial incentives for smoking cessation and breastfeeding when discussed in relation to the Tappin et al. [34] and Heil et al. [39] intervention vignettes were considered to have ‘appeal’ particularly amongst teenage mothers and/or families ‘where money is an issue’ to ‘attract’ or ‘persuade’ them to engage in health services and for a ‘meaningful conversation’ to enable them to make ‘informed choices’ regarding their health behaviours:

I think if you were young, or if you were on your own and you might feel a big judged at times, or a bit….you know, why’s this person coming to look at me again, you know and I think the incentive scheme can only kind of help that really and make it a nicer experience (T9, I, mother). (Thomson et al., 2014)

### CMOC4
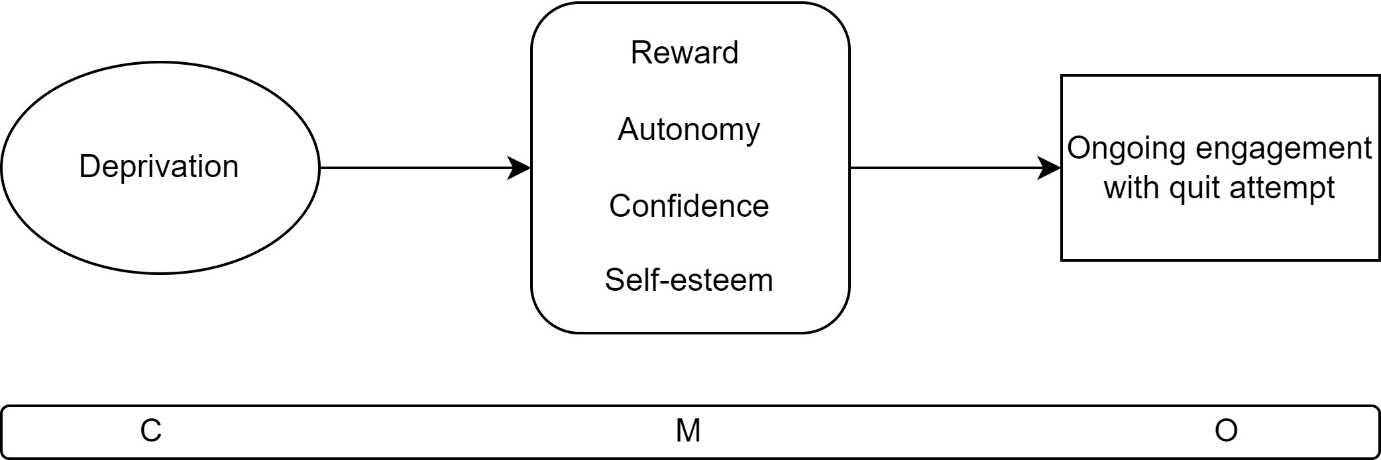


For people who smoke and come from a deprived background it is common to internalise societal messages which blame individual behaviour for poor health outcomes which are, at least in part, caused by structural conditions such as poverty (C). In such a context, financial incentives for giving up smoking act as a reward and promotes feelings of autonomy, confidence, self-esteem, and respect in people who otherwise do not often feel valued or seen (M). As a result they are more likely to make a stop smoking attempt and to keep engaging with services (O).

Participants were asked how they felt about the incentive. Most participants liked and appreciated the vouchers as a reward for success in quitting. ‘I like it; I’ve been using it to do fun things, too’ (participant 17, successful quitter, moderate income). (Van den Brand et al., 2018)

Reasons to Enroll in Incentive Programs

The most common reason participants would consider enrolling was because the money would affect the individual’s life (43.54%), followed by the money compensating how hard it would be to quit (32.65%), and being paid signaling the importance of quitting (14.97%). (Breen, Ferguson, et al., 2021)

Women felt that the effort they put into behaviour change deserved recognition and validation: ‘Yeah, I think it has to be for you because you're the one that's doing it, no one else is'. One woman who had been part of an incentive programme for breastfeeding remarked on the ‘really well thought out nice gifts’. It appears that this participant inferred some thoughtful deliberation behind the choice of incentives used, and consequently according them signiﬁcance beyond their ﬁnancial value. (Crossland et al., 2015)

Our ﬁndings suggest that incentives may be more effective if they offer autonomy, pleasure and convey esteem. (Crossland et al., 2015)

The latter possibility highlights the role of various social and physical environmental factors in the reduced successes of those who are deprived that were not assessed in the current study. These include a lack of social support in quitting among those who are more deprived [24], increased exposure to smoking (with attendant inﬂuence on social norms and mirror neurones), given the higher prevalence of smoking among socially deprived groups, and the possible increased density of tobacco retailers in areas of higher deprivation, which can reduce the success of quitting [29]. (Ierfino et al., 2015)

Incentivised women appeared to be using the services more as a result of the incentives. This greater engagement may have given women in this group more of an opportunity to experience service related support. Furthermore, because voucher delivery was contingent upon biochemically confirmed smoking cessation, monitoring of smoking behaviour and provision of related feedback from the services might have been more regular for incentivised women. (Mantzari et al., 2012)

Among those who felt it had been a factor in encouraging them to stay quit, the incentive was viewed as: a ‘reward’, ‘bonus’ or ‘wee treat’ for quitting; a ‘wee bit extra to keep you going’ with the quit attempt; providing ‘something to work towards’; and a reason to stick with the programme or to keep going back to the pharmacist.

‘I was going to stop anyways. (It was) just a, certainly a bonus having that there like. I reckon it would probably encourage you to keep going back to the chemist.’ (P209, quit)

Similar views were expressed by service providers, who suggested that the incentive encouraged participants to stick with formal support for longer, which in turn increased their chances of quitting successfully. It was also suggested that the ﬁnancial incentive might encourage participants to come back for support, even if they relapsed.

‘It’s also an incentive to come back as well to the Pharmacy, even if they have sort of failed in the ﬁrst couple of weeks – again, you just use that to actually reinforce that “OK, it’s difﬁcult to start with, but there’s also £12.50 a week if you do sort of come a bit better towards the end.’ (Pharmacist 1) (Ormston et al., 2015)

Breadline survivors. These were mothers who appeared to be the most socially and financially disadvantaged, typically single mothers and/or with unsupportive partners living in impoverished circumstances. They tended to place a particularly high value on the financial rewards offered by the scheme which were more likely to be used for buying staples such as groceries rather than baby products or treats. Mothers in this group tended to have relatively low self-esteem and confidence in their ability to stop. (Radley et al., 2013)

Financial incentives for smoking cessation and breastfeeding when discussed in relation to the Tappin et al. [34] and Heil et al. [39] intervention vignettes were considered to have ‘appeal’ particularly amongst teenage mothers and/or families ‘where money is an issue’ to ‘attract’ or ‘persuade’ them to engage in health services and for a ‘meaningful conversation’ to enable them to make ‘informed choices’ regarding their health behaviours:

I think if you were young, or if you were on your own and you might feel a big judged at times, or a bit….you know, why’s this person coming to look at me again, you know and I think the incentive scheme can only kind of help that really and make it a nicer experience (T9, I, mother). (Thomson et al., 2014)

A few professionals reflected that incentives would provide vulnerable individuals with one of the first opportunities to receive a reward and acknowledgement for an achievement. Unrestrictive incentives like shopping vouchers could provide the most disadvantaged families with a rare opportunity for autonomy to ‘make decisions about what they ought to be spending the additional money on’, such as providing ‘treats’ for themselves and their families. (Thomson et al., 2014)

Autonomy is of particular note where incentives are perceived as bribes which can undermine free will and reflect a ‘nanny state’ resulting in diminished individual responsibility for health choices [58]. In contrast, consumers and professionals in our study reported that unrestricted vouchers can promote individual autonomy for the most disadvantaged through providing a rare opportunity for choice and self-reward. Similarly discourses of incentives as ‘unfair’ for rewarding ‘bad behaviour’, of discouraging individual responsibility or for targeting only disadvantaged communities contrast with narratives of feeling valued, more confident and improved self-esteem. Our interpretation is that media debates on the advantages and disadvantages of financial incentives can be easily biased towards intellectual philosophical, political and ethical arguments about the role of the state, without considering the perspectives of more disadvantaged families who are struggling to do the best for their children. This is particularly important as children have no choice and public acceptability is greatest for incentives to protect their wellbeing [2]. (Thomson et al., 2014)

### CMOC5


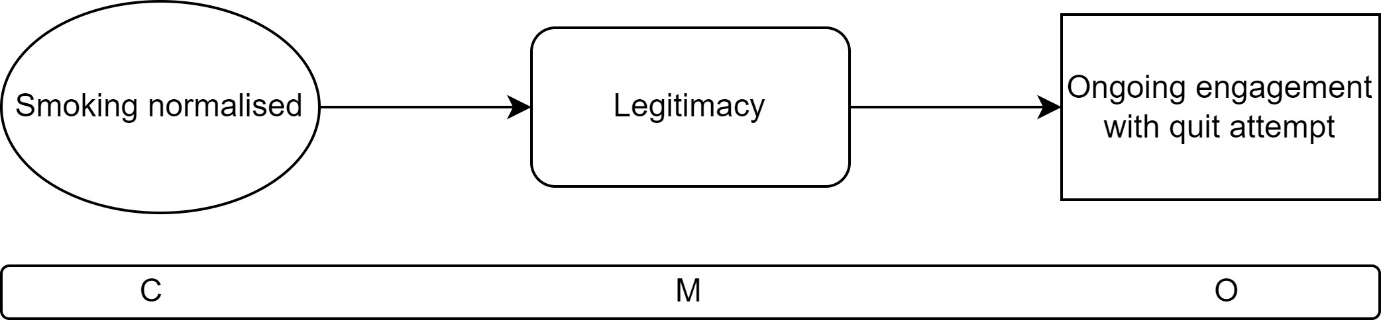


Where a social network and peer group normalises smoking (C), a financial incentive legitimises a quit attempt, providing cover for other reasons which may be less accepted within the peer group (M) and as a result a quite attempt is more likely (O).

Incentives can also promote connections within women's existing social networks, and such interventions show promise (Morgan et al., in press). Social context is important for smoking (Graham et al., 2012) and infant feeding (Brown et al., 2011) with partners, family and community playing inﬂuential roles in women's decision making and capacity for behaviour change. Hedonic incentives beyond the individual such as friend and family activities can enhance wellbeing, and could help women enlist the help of signiﬁcant others. (Crossland et al., 2015)

The latter possibility highlights the role of various social and physical environmental factors in the reduced successes of those who are deprived that were not assessed in the current study. These include a lack of social support in quitting among those who are more deprived [24], increased exposure to smoking (with attendant inﬂuence on social norms and mirror neurones), given the higher prevalence of smoking among socially deprived groups, and the possible increased density of tobacco retailers in areas of higher deprivation, which can reduce the success of quitting [29]. (Ierfino et al., 2015)

My girlfriend hasn’t helped much considering she asks me if I wanna [sic] smoke one when she gets home everyday [sic]. [Male, 21 years] (McKelvey & Ramo, 2018)

If the incentive is presented in a private context within a controlled environment (e.g., randomized clinical trials), social influences, such as informal social control or social image processes, are limited. Alternatively, incentives offered in more public settings can activate social influences (Gneezy et al., 2011). Public settings are marked by the presence of others, and this can influence individuals’ behavior by inspiring social responses to incentives instead of strictly monetary responses. For example, social reactions such as interpersonal discussions about the incentive can activate behavior based on social image maintenance or prevailing social norms. (Parks et al., 2016)

Although participants felt that smokers could understand the difficulty of quitting, they did not find current smokers supportive of their quit attempt and felt that they undermined their attempts to quit.

My best friend, when I was trying to quit everyday she would say ‘are you really quitting? You’re really quitting? You don’t want a cigarette?’ I think there’s a guilt thing about being a smoker that you want other people to join in with you. I’m not the only one being a little naughty. (Thomas et al., 2015)

Participants felt that often the people they told about their decision to quit smoking did not believe they could do it. This pertained to both smokers and non-smokers. The perceived lack of faith in their ability to quit, sometimes translated into negative outcomes. For example, one participant did not even try to quit after her friends doubted her.

… My friends were like you can’t do it. Not that they ever said that but it was like kind of the impression that they gave off. So then I was like alright, whatever, then I won’t do it. (Thomas et al., 2015)

For geographically targeted incentives, some participants expressed concerns towards a ‘postcode lottery’ of care and believed ‘equity’ to be important as everyone needs support. However, universal incentives could ‘benefit those who already had enough’. Some professionals believed that targeted provision could help address embedded ‘social norms’ associated with the target behaviours. Many participants considered how incentives had the potential to reduce inequalities through providing access to items that they could not afford (e.g. breast pump, nursing bras), and financial support for those who are ‘struggling for money’ to buy essentials such as ‘food’, ‘things for the baby’ or for a ‘healthier lifestyle’ (Thomson et al., 2014)

Money can certainly provide an incentive to take on a new form of activity. But it can also operate in another way; it can give ‘argumentative cover’ to allow one to justify a form of action to one’s peers, especially when the real reason is one that either they would not accept or would be hard to offer in the circumstances. (Wolff, 2015)

I suggested that we are often expected to give reasons to our peers to explain and justify our behaviour, and that this will be especially so when our behaviour is outside of the norms of our peer group. Critically, however, the real reason for performing an action may not be a reason one would chose to give to one’s peer group, but another reason might give ‘argumentative cover’ in that it is acceptable within the peer group. (Wolff, 2015)

Hence, I believe it is possible that paying people to do things need not, strictly speaking, act as an incentive as economists typically understand that notion, but can act as a rationalization, allowing an individual to justify his or her behaviour to a peer group that would not otherwise accept the motivation as a justifying reason. On this understanding, cash payments allow individuals to act on non-cash motivations they already have. Under these circumstances, payments are not corrupting. On the contrary, they can be liberating. (Wolff, 2015)

### CMOC6


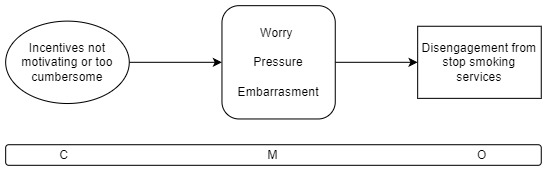


Where service users are not motivated by financial incentives or where they find the requirements needed to receive the incentive too cumbersome, not worth the effort, or in conflict with other life tasks and commitments (C), participants may feel unmotivated or may worry how they will be perceived if they fall short of quitting (M) and as a result may disengage from the programme (O).

Reasons for disengagement hinted at a felt change in clients’ relationship with service providers, with the incentive introducing a quasi-contractual relationship. This placed the patient in the role of “providing the service” (smoking cessation) and the health care professional as the “buyer”. For some, this relationship manifested itself in a sense of obligation to the service providers, manifest most clearly when they had “failed to deliver”:

“.. ..I just felt I had let them down as well. Even though it is yourself, you still feel as if you are letting other people down as well which again is a horrible feeling so then you feel guilt again and I think, I’m just gonna have a fag.” (female, 35, lab worker, group 3) (Allan et al., 2012)

Clients also highlighted the impact of the administrative burden. Locality of monitoring, individual/peer meetings, paperwork and timing of monitoring related to convenience and raised issues such as childcare, working hours and meals times. The threshold of what was perceived as inconvenient varied between individuals. Perceived costs and barriers have been implicated in a number of behavioural theories attempting to explain and predict behaviour.25 Solutions may focus on readdressing the balance between perceived costs and beneﬁts. In an incentive based scheme this may point to increasing the value of the reward. However, not only would this make the scheme more costly, it may also increase felt obligation and guilt on relapse, thereby inhibiting re-engagement. As a result, it may be more cost-effective to address the problem by reducing barriers/costs such as inconvenience, lack of privacy, timing and embarrassment of association with methadone use. (Allan et al., 2012)

. . some participants stopped engaging in the program once they had achieved their own goal of quitting smoking (this could not be verified as they had stopped engaging in the program and providing CO samples) and returning to the pharmacy for further checkins was deemed unnecessary or inconvenient (eg due to distance or timing). These time, distance or convenience factors might be barriers to sustained engagement which will disproportionately influence individuals in more remote localities.

Further steps could be taken to ensure sustained engagement. As suggested by participants within post- program interviews, this could include providing check- ins weekly for the entire program. Alternative measures to increase the flexibility or convenience of the program could also be explored, including Internet-based programs.(Breen, Frandsen, et al., 2021)

It also appeared to have arisen from their fear of being told off for not trying to quit: “So I was constantly thinking about keeping my carbon monoxide levels down so I don’t get into trouble... I thought it was like I keep smoking like my five/six a day then my carbon monoxide levels will either stay the same or go up a little bit. And it would be like, “You’re not trying to quit why should I bother with you because you’re not even participating”. Do you know what I mean?” (Participant20, incentivised group) (Mantzari et al., 2012)

She was okay (the pharmacist), but I think at the same time when I did fall back and I said to her ‘I’ve had one extra than I’m supposed to have today.’ She was kind of like ‘oh no that’s just bad’, she wasn’t sort of sympathetic, if you like. So I just says to her ‘well when you stopped smoking did you not fall back and have one?’ And she said ‘yeah’ and I went ‘well don’t judge me! It was just an accident, a mistake.’ (Radley et al., 2013)

[Participant quote:] ‘I was at the chemist seeing if I could get patches without having to go to the doctor and she took me in to this little room and gave me loads of leaflets and one of them was about this baby thing, I wasn’t really too bothered about it but she put my name down and everything anyway because I wasn’t sure at the time whether I’d be able to stick to it or not so, but that’s how I heard about it. (.. .) She asked me if I wanted to go for it or not and I kind of felt obliged to because I was just in this wee room and there wasn’t much going on, there was all these leaflets in front of me and I just kind of, I mean I only went in there to get patches and I was getting hit with all this stuff and I was like ‘Just put my name down, whatever’ (Radley et al., 2013)

Cattaneo et al intervention vignette [43] reported that provider incentives could lead to women ‘feeling bombarded’, ‘bullied’ or ‘inappropriately handled’ in attempts to ‘manipulate people into a particular behaviour’; which in turn could exacerbate unhealthy behaviours, i.e. women ‘smoking even more’ or being less inclined to breastfeed. Other concerns were how providers may incorporate ‘bias and opinions rather than research and fact’ and provider–women interactions relegated to a ‘tick-box’ exercise. (Thomson et al., 2014)

The potential positive and negative impact of incentive schemes on provider–women relationships in our study are evident in the literature: increased access and improved rapport [29]; mistrust and alienation [4,30] and limiting support for non-target behaviours [4,5]. These findings highlight the need for sensitive, authentic, person-centred communication [61] as women dislike feeling judged or pressurised to behave in a way deemed appropriate by others [54,62]. (Thomson et al., 2014)

## Bibliography

Allan, C., Radley, A., & Williams, B. (2012). Paying the price for an incentive: an exploratory study of smokers' reasons for failing to complete an incentive based smoking cessation scheme. *Journal of health services research & policy*, *17*(4), 212-218. <https://doi.org/https://dx.doi.org/10.1258/jhsrp.2012.011084>

Bisaga, A., Padilla, M., Garawi, F., Sullivan, M. A., & Haney, M. (2007). Effects of alternative reinforcer and craving on the choice to smoke cigarettes in the laboratory. *Human Psychopharmacology: Clinical and Experimental*, *22*(1), 41-47. <https://doi.org/10.1002/hup.816>

Breen, R. J., Ferguson, S. G., & Palmer, M. A. (2021). Smokers' Perceptions of Incentivized Smoking Cessation Programs: Examining How Payment Thresholds Change With Income. *Nicotine & tobacco research : official journal of the Society for Research on Nicotine and Tobacco*, *23*(9), 1567-1574. <https://doi.org/https://dx.doi.org/10.1093/ntr/ntab031>

Breen, R. J., Frandsen, M., & Ferguson, S. G. (2021). Incentives for smoking cessation in a rural pharmacy setting: The Tobacco Free Communities program. *The Australian journal of rural health*, *29*(3), 455-463. <https://doi.org/https://dx.doi.org/10.1111/ajr.12724>

Breen, R. J., Palmer, M. A., Frandsen, M., & Ferguson, S. G. (2022). Design of financial incentive programmes for smoking cessation: A discrete choice experiment. *Nicotine & tobacco research : official journal of the Society for Research on Nicotine and Tobacco*(drz, 9815751). <https://doi.org/https://dx.doi.org/10.1093/ntr/ntac042>

Chivers, L. L., Higgins, S. T., Heil, S. H., Proskin, R. W., & Thomas, C. S. (2008). Effects of initial abstinence and programmed lapses on the relative reinforcing effects of cigarette smoking. *Journal of Applied Behavior Analysis*, *41*(4), 481-497. <https://doi.org/10.1901/jaba.2008.41-481>

Crossland, N., Thomson, G., Morgan, H., Dombrowski, S. U., Hoddinott, P., & team, B. s. (2015). Incentives for breastfeeding and for smoking cessation in pregnancy: an exploration of types and meanings. *Social science & medicine (1982)*, *128*(ut9, 8303205), 10-17. <https://doi.org/https://dx.doi.org/10.1016/j.socscimed.2014.12.019>

Higgins, S. T., Washio, Y., Heil, S. H., Solomon, L. J., Gaalema, D. E., Higgins, T. M., & Bernstein, I. M. (2012). Financial incentives for smoking cessation among pregnant and newly postpartum women. *Preventive Medicine*, *55*(SUPPL.), S33-S40. <https://doi.org/10.1016/j.ypmed.2011.12.016>

Ierfino, D., Mantzari, E., Hirst, J., Jones, T., Aveyard, P., & Marteau, T. M. (2015). Financial incentives for smoking cessation in pregnancy: a single-arm intervention study assessing cessation and gaming. *Addiction (Abingdon, England)*, *110*(4), 680-688. <https://doi.org/https://dx.doi.org/10.1111/add.12817>

Ladapo, J. A., Tseng, C.-H., & Sherman, S. E. (2020). Financial Incentives for Smoking Cessation in Hospitalized Patients: A Randomized Clinical Trial. *The American journal of medicine*, *133*(6), 741-749. <https://doi.org/https://dx.doi.org/10.1016/j.amjmed.2019.12.025>

Mantzari, E., Vogt, F., & Marteau, T. M. (2012). The effectiveness of financial incentives for smoking cessation during pregnancy: is it from being paid or from the extra aid? *BMC pregnancy and childbirth*, *12*(100967799), 24. <https://doi.org/https://dx.doi.org/10.1186/1471-2393-12-24>

McKelvey, K., & Ramo, D. (2018). Conversation Within a Facebook Smoking Cessation Intervention Trial For Young Adults (Tobacco Status Project): Qualitative Analysis. *JMIR formative research*, *2*(2), e11138. <https://doi.org/https://dx.doi.org/10.2196/11138>

Meredith, S. E., Jarvis, B. P., Raiff, B. R., Rojewski, A. M., Kurti, A., Cassidy, R. N., Erb, P., Sy, J. R., & Dallery, J. (2014). The ABCs of incentive-based treatment in health care: a behavior analytic framework to inform research and practice. *Psychology research and behavior management*, *7*(101514563), 103-114. <https://doi.org/https://dx.doi.org/10.2147/PRBM.S59792>

Notley, C., Gentry, S., Livingstone-Banks, J., Bauld, L., Perera, R., & Hartmann-Boyce, J. (2019). Incentives for smoking cessation. *The Cochrane database of systematic reviews*, *7*(100909747), CD004307. <https://doi.org/https://dx.doi.org/10.1002/14651858.CD004307.pub6>

Ormston, R., van der Pol, M., Ludbrook, A., McConville, S., & Amos, A. (2015). quit4u: the effectiveness of combining behavioural support, pharmacotherapy and financial incentives to support smoking cessation. *Health education research*, *30*(1), 121-133. <https://doi.org/https://dx.doi.org/10.1093/her/cyu024>

Parks, M. J., Slater, J. S., Rothman, A. J., & Nelson, C. L. (2016). Interpersonal Communication and Smoking Cessation in the Context of an Incentive-Based Program: Survey Evidence From a Telehealth Intervention in a Low-Income Population. *Journal of health communication*, *21*(1), 125-133. <https://doi.org/https://dx.doi.org/10.1080/10810730.2015.1039677>

Radley, A., Ballard, P., Eadie, D., MacAskill, S., Donnelly, L., & Tappin, D. (2013). Give It Up For Baby: outcomes and factors influencing uptake of a pilot smoking cessation incentive scheme for pregnant women. *BMC public health*, *13*(100968562), 343. <https://doi.org/https://dx.doi.org/10.1186/1471-2458-13-343>

Thomas, J. L., Bengtson, J. E., Ghidei, W., Schreier, M., Wang, Q., Luo, X., Lust, K., & Ahluwalia, J. S. (2015). Social contingencies and college Quit and Win contest: A qualitative inquiry. *American Journal of Health Behavior*, *39*(2), 231-240. <https://doi.org/10.5993/AJHB.39.2.10>

Thomson, G., Morgan, H., Crossland, N., Bauld, L., Dykes, F., Hoddinott, P., team, B., Dombrowski, S., MacLennan, G., Rothnie, K., Stewart, F., Farrar, S., Yi, D., Hislop, J., Ludbrook, A., Campbell, M., Moran, V. H., Sniehotta, F., & Tappin, D. (2014). Unintended consequences of incentive provision for behaviour change and maintenance around childbirth. *PloS one*, *9*(10), e111322. <https://doi.org/https://dx.doi.org/10.1371/journal.pone.0111322>

van den Brand, F. A., Candel, M. J. J. M., Nagelhout, G. E., Winkens, B., & van Schayck, C. P. (2021). How Financial Incentives Increase Smoking Cessation: A Two-Level Path Analysis. *Nicotine & tobacco research : official journal of the Society for Research on Nicotine and Tobacco*, *23*(1), 99-106. <https://doi.org/https://dx.doi.org/10.1093/ntr/ntaa024>

Van den Brand, F. A., Dohmen, L. M. E., Van Schayck, O. C. P., & Nagelhout, G. E. (2018). 'Secretly, it's a competition': a qualitative study investigating what helped employees quit smoking during a workplace smoking cessation group training programme with incentives. *BMJ Open*, *8*(11), e023917. <https://doi.org/https://dx.doi.org/10.1136/bmjopen-2018-023917>

Wolff, J. (2015). Paying people to act in their own interests: Incentives versus rationalization in public health. *Public Health Ethics*, *8*(1), 27-30. <https://doi.org/10.1093/phe/phu035>
