# Supplementary material for: How and why do financial incentives contribute to helping people stop smoking? A realist review
Source: BMC Public Health. 2024 Feb 16;24:500. doi: 10.1186/s12889-024-17967-3 (PMC10873947; doi:10.1186/s12889-024-17967-3)
Supplement: Supplementary file 2 — Supplementary Material 2 [file 12889_2024_17967_MOESM2_ESM.pdf]

Authors	Year	Title	Country	Population	Intervention/experiment	Study design	Results
Allan C, Radley A, Williams B.	2012	Paying the price for an incentive: an exploratory study of smokers' reasons for failing to complete an incentive based smoking cessation scheme.	Scotland	Participants in Quit4u stop smoking programme who had not successfully completed the programme	Quit4u stop smoking intervention targeting people living in areas of deprivation. Financial incentive amount was £12.50 weekly in supermarket vouchers, for up to 12 weeks.	Qualitative study based on 14 in-depth interviews	Study explores the factors that contribute to what goes wrong when financial incentives do not work.
Bisaga A, Padilla M, Garawi F, Sullivan MA, Haney M.	2007	Effects of alternative reinforcer and craving on the choice to smoke cigarettes in the laboratory.	USA	Current smokers not currently seeking to stop	Laboratory study examining the extent to which smokers were willing to choose various amounts of money over puffs on a cigarette.	After abstaining from tobacco use over night, participants were presented, five times with ten minutes between each, with choices of puffs on cigarettes or money ranging form $0.50-3. Craving was also measured using validated instrument.	The probability of study participants choosing to smoke decreased significantly with the increase in the magnitude of the financial incentive.
Breen RJ, Ferguson SG, Palmer MA.	2021	Smokers' Perceptions of Incentivized Smoking Cessation Programs: Examining How Payment Thresholds Change With Income	UK	Current smokers. Three income groups represented: low, middle and high income earners. (n=203)	Survey presenting hypothetical stop smoking programme to current smokers assessing the level of financial incentive they would require to stop smoking in return. Incentive amounts were: £50–75 per week for ten weeks (£500–£750 total).	Online survey presenting various scenarios. Incentive amounts were: £50–75 per week for ten weeks (£500–£750 total).	Increasing incentive amounts corresponded to increasing interest in undertaking the stop smoking programme up to £50–75 per week (£500–£750 total). Amounts above these did not significantly increase interest in the intervention. People on a high income found amounts less motivating than those on a low income, but likelihood of enrolment was the same across income groups.
Breen RJ, Frandsen M, Ferguson SG.	2021	Incentives for smoking cessation in a rural pharmacy setting: The Tobacco Free Communities program.	Australia	Adult smokers (n=62)	Pharmacy-led financial incentive program for smoking cessation. Incentive amounts were: £50–75 per week for ten weeks (£500–£750 total).	Multi- site single- arm trial.	The intervention was found to be feasible. 19.35% of participants were abstinent at the end of the study.
Breen RJ, Palmer MA, Frandsen M, Ferguson SG.	2022	Design of financial incentive programmes for smoking cessation: A discrete choice experiment.	UK	Current smokers (n=430)	Choice given of two hypothetical scenarios comprising of five options related to incentive amount and type, frequency and schedule, and programme location. Hypothetical amounts: £200 over ten weeks vs £300 over ten weeks.	Online discreet choice experiment.	Participants preferred: weekly sessions in a healthcare setting with higher amounts over lower; cash over vouchers; and consistent amounts over an escalating schedule.

Chivers LL, Higgins ST, Heil SH, Proskin RW, Thomas CS.	2008	Effects of initial abstinence and programmed lapses on the relative reinforcing effects of cigarette smoking.	USA	Adult smokers (n=58)	Laboratory study to analyse the association between brief smoking lapses and the risk of relapse in people who have stopped smoking. Incentive amount: $3 the first time the participant presented with a CO sample indicating abstinence. The amount rose by $0.50 for each subsequent consecutive sample up to a maximum of $10 where it remained for each negative sample thereafter. Additionally, a $10 bonus was given for each three consecutive negative CO samples	Participants were paid an incentive to remain abstinent for 14 days at the start of the study. After the 14 days they were assigned randomly to one of four scenarios where they smoked 0 puffs, 1 puff or 8 puffs. Financial incentives were attached to the various scenarios and payment was withheld when people did not meet required abstinence criterion.	Programmed lapses in smoking abstinence did not disrupt stop smoking attempts. The effect of financial incentives was positive.
Crossland N, Thomson G, Morgan H, Dombrowski SU, Hoddinott P, team Bs.	2015	Incentives for breastfeeding and for smoking cessation in pregnancy: an exploration of types and meanings.	UK	Pregnant women; Service providers; Experts/decision makers	Typology of incentives and their meanings for stopping smoking during pregnancy and breastfeeding.	Two systematic reviews and primary qualitative data collection. Interview and focus group data were collected and analysed using the Framework method.	Financial incentives for stopping smoking are complex and can be understood in three dimensions which influence their meaning to the recipient: degree of restriction; whether they are hedonistic or utilitarian; the value of the money.
Higgins ST, Washio Y, Heil SH, Solomon LJ, Gaalema DE, Higgins TM, et al.	2012	Financial incentives for smoking cessation among pregnant and newly postpartum women.	USA	Economically disadvantaged pregnant women who smoke.	Six trials on the use of financial incentives with women who smoke and are pregnant. Incentives given are in the range $25-50 per month.	Synthesis to combine the six studies which were undertaken by same authors	Financial incentives resulted in more successful stopping smoking efforts than other methods and authors conclude that they may be needed for populations like deprived women who smoke heavily in order to encourage them to successfully engage with a stop smoking attempt.
Ierfino D, Mantzari E, Hirst J, Jones T, Aveyard P, Marteau TM.	2015	Financial incentives for smoking cessation in pregnancy: a single-arm intervention study assessing cessation and gaming.	England	Pregnant women who smoke living in high deprivation area	Pregnant women (n=239) attending English hospital were enrolled 12-week stopping smoking course with nicotine replacement and financial incentives. The scheme involved provision of a shopping voucher upon CO validation of self-reported abstinence. The size of incentives increased by £1 for each visit at which smoking cessation was confirmed, from the first voucher (worth £8) to the last voucher (worth £39), providing a maximum total of £752-worth of vouchers.	Single-arm intervention study	Higher quit rates in incentivised cohort compared with historical controls from same hospital.

Ladapo JA, Tseng C-H, Sherman SE.	2020	Financial Incentives for Smoking Cessation in Hospitalized Patients: A Randomized Clinical Trial.	USA	Low-income, currently smoking, hospitalised people attending Veterans Affairs (VA) hospital (n=182)	Comparison of financial incentives plus enhanced usual care versus enhanced usual care alone (providing stop smoking therapy including referral to quit line, education, counselling and pharmacotherapy). Financial incentives were attached to each component and participants could earn up to $550 for participation all components and if keeping abstinent at 2 and 6 months.	Randomised controlled trial	At 6 months after hospital discharge, there was a non-significant higher stopping smoking rates among patients randomised to financial incentives as opposed to usual care.
Mantzari E, Vogt F, Marteau TM.	2012	The effectiveness of financial incentives for smoking cessation during pregnancy: is it from being paid or from the extra aid?	UK	Pregnant women who smoke (n=36)	Standard NHS stop smoking services with (n=20) and without (n=16) additional financial incentives	Qualitative study based on 36 in-depth interviews. Analysis used Framework Analysis	Incentivised group used services more and felt more motivated due to increased monitoring and feedback than non-incentivised group.
McKelvey K, Ramo D.	2018	Conversation Within a Facebook Smoking Cessation Intervention Trial For Young Adults (Tobacco Status Project): Qualitative Analysis.	USA	Young adults who smoke (n=138)	Stop smoking group intervention featuring message board in Facebook. Incentive amount: up to $90 for posting in Facebook group.	Qualitative analysis of Facebook messages	Four dominant themes: coping skills; friends and family; motivation to quit; benefits of quitting
Meredith SE, Jarvis BP, Raiff BR, Rojewski AM, Kurti A, Cassidy RN, et al.	2014	The ABCs of incentive-based treatment in health care: a behaviour analytic framework to inform research and practice.	USA	N/A	Guidance for treatment providers for designing and implementing incentive-based interventions that promote healthy behaviour in a number of domains.	Integrated review of published literature and theory	N/A
Notley C, Gentry S, Livingstone-Banks J, Bauld L, Perera R, Hartmann-Boyce J.	2019	Incentives for smoking cessation. The Cochrane database of systematic reviews.	USA, Thailand, Philippines, Europe.	Current smokers (n=excess of 21,6000)	Different incentivised stop smoking trials. Financial incentive amounts: USD 45-USD 1185.	Cochrane review of randomised controlled trials	33 studies were included. The pooled risk ratio for stopping smoking with incentives at longest follow up (6 months +) was 1.49 compared with controls.
Ormston R, van der Pol M, Ludbrook A, McConville S, Amos A.	2015	quit4u: the effectiveness of combining behavioural support, pharmacotherapy and financial incentives to support smoking cessation.	Scotland	Adult smokers living in deprived areas	Quit4u stop smoking intervention combining stop smoking services with financial incentives. Additional survey and interview studies were undertaken to examine the experiences and vies of services users and professionals. Financial incentive amount was £12.50 weekly in supermarket vouchers, for up to 12 weeks.	Quasi-experimental study using routine data comparing intervention with regular stop smoking services in other locations in Scotland	Quit4u had higher stop smoking rates than other stop smoking services in Scotland. Successful features of the programme were: one-to-one pharmacy encouragement and support along with repeated CO tests, cessation groups, and pharmacotherapy.

Parks MJ, Slater JS, Rothman AJ, Nelson CL.	2016	Interpersonal Communication and Smoking Cessation in the Context of an Incentive-Based Program: Survey Evidence From a Telehealth Intervention in a Low-Income Population.	USA	Low-income people who smoke (n=970)	Population-level telehealth intervention offered a small incentive ($20) participation in intervention consisting of quitline telephone counselling as well as offers of self-help materials and pharmacotherapy.	Survey at least 7 months after initial enrolment in the study.	Study participants were more likely to use quitline and undertake behavioural steps associated with both short-term and long-term stop smoking success.
Radley A, Ballard P, Eadie D, MacAskill S, Donnelly L, Tappin D.	2013	Give It Up For Baby: outcomes and factors influencing uptake of a pilot smoking cessation incentive scheme for pregnant women.	Scotland	Pregnant women who smoke (n=393)	Women who chose to participate set a quit date and received an initial short intervention where they were offered pharmacotherapy. They then attended for weekly support sessions at their local pharmacy for up to 12 weeks where their abstinence was verified using a carbon monoxide breath test. Financial incentive amount was £12.50 in weekly supermarket vouchers, for up to 12 weeks.	Multi-methods evaluation of smoking related outcomes and the experience of participating in the intervention	Higher engagement and stop smoking rates than other non-incentive based stop smoking interventions for pregnant women in Scotland. Further a typology was developed showing factors which contributed to the success, or lack there of, of the financial intervention in various population groups.
Thomas JL, Bengtson JE, Ghidei W, Schreier M, Wang Q, Luo X, et al.	2015	Social contingencies and college Quit and Win contest: A qualitative inquiry.	USA	University students (n=27) who smoke	Quit and Win contest in a university setting. Financial incentive was 'smaller gift cards' (no amount given) and the chance to win a trip for 2 to the Caribbean worth $3000 or the equivalent in retail gift cards.	Data were colleted via six focus groups regarding motivations to join the intervention, reasons for wanting to stop smoking, disclosing the decision to quit, reactions from others, social support, and triggers for relapse.	Participants joined the study because the received regular gift cards (rather than for the chance to win a few bigger prizes) and because they felt it would provide the extra motivation they needed to quit. Participants were reluctant to disclose their stop smoking attempts to friends and family for fear that they would fail and be judged.
Thomson G, Morgan H, Crossland N, Bauld L, Dykes F, Hoddinott P, et al.	2014	Unintended consequences of incentive provision for behaviour change and maintenance around childbirth.	UK	Pregnant women or recent mothers who smoke, as well as their partners and other family members; service providers; experts/decision makers	Aim of study was to understand participants' knowledge, experiences and attitudes toward the use of financial incentives, particularly in relation to the positive and negative unintended consequences of strategies to help pregnant women and recent mothers to stop smoking.	Multi-site mixed methods study using evidence synthesis, focus groups, interviews, and surveys	Four key themes: how incentives can increase or diminish inequalities; enhance or lessen intrinsic motivation and wellbeing; have a positive or negative effect on relationships within personal networks or with health providers; and can impact on health systems and resources.

van den Brand FA, Candel MJJM, Nagelhout GE, Winkens B, van Schayck CP.	2021	How Financial Incentives Increase Smoking Cessation: A Two-Level Path Analysis.	Netherlands	Tobacco smoking employees from 61 companies	Stop smoking group counselling in the work place. Vouchers were distributed to intervention group upon abstinence directly after finishing the smoking cessation program (€50), after three months (€50), after six months (€50), and after 12 months (€200). Participants in the control group did not receive incentives.	Secondary data analysis using two-level path analysis of data collected from a cluster randomised trial where intervention group received €350 in total for 12 months continuous smoking abstinence.	Financial incentives were associated with higher use of pharmacotherapy and with a higher stop smoking success. The intervention was viewed more positively by participants with a higher degree of self-efficacy, more social influence to quit and with a positive attitude about quitting.
Van den Brand FA, Dohmen LME, Van Schayck OCP, Nagelhout GE.	2018	Secretly, it's a competition': a qualitative study investigating what helped employees quit smoking during a workplace smoking cessation group training programme with incentives.	Netherlands	Tobacco smoking employees from nine companies	Stop smoking group counselling in the work place. Vouchers were distributed to intervention group upon abstinence directly after finishing the smoking cessation program (€50), after three months (€50), after six months (€50), and after 12 months (€200). Participants	Semi-structured qualitative interviews with people who had successfully and unsuccessfully stopped smoking during a workplace cluster randomised controlled trial in which a group intervention was provided which included financial incentives	Important factors which contributed to stop smoking success according to interviewees were the workplace cessation programme, personal motivation and peer support, but not the incentives.
Wolff J.	2015	Paying people to act in their own interests: Incentives versus rationalization in public health.	UK	N/A	N/A	Editorial type article which discusses	N/A
